# USP7 Is a Tumor-Specific WNT Activator for *APC*-Mutated Colorectal Cancer by Mediating β-Catenin Deubiquitination

**DOI:** 10.1016/j.celrep.2017.09.072

**Published:** 2017-10-17

**Authors:** Laura Novellasdemunt, Valentina Foglizzo, Laura Cuadrado, Pedro Antas, Anna Kucharska, Vesela Encheva, Ambrosius P. Snijders, Vivian S.W. Li

**Affiliations:** 1 The Francis Crick Institute, 1 Midland Road, London NW1 1AT, UK; 2 The Francis Crick Institute, Mass Spectrometry Science Technology Platform, 1 Midland Road, London NW1 1AT, UK

**Keywords:** Wnt signaling, APC, β-catenin, ubiquitination, USP7, colorectal cancer

## Abstract

The tumor suppressor gene adenomatous polyposis coli (APC) is mutated in most colorectal cancers (CRCs), resulting in constitutive Wnt activation. To understand the Wnt-activating mechanism of the *APC* mutation, we applied CRISPR/Cas9 technology to engineer various APC-truncated isogenic lines. We find that the β-catenin inhibitory domain (CID) in APC represents the threshold for pathological levels of Wnt activation and tumor transformation. Mechanistically, CID-deleted APC truncation promotes β-catenin deubiquitination through reverse binding of β-TrCP and USP7 to the destruction complex. USP7 depletion in *APC*-mutated CRC inhibits Wnt activation by restoring β-catenin ubiquitination, drives differentiation, and suppresses xenograft tumor growth. Finally, the Wnt-activating role of USP7 is specific to *APC* mutations; thus, it can be used as a tumor-specific therapeutic target for most CRCs.

## Highlights

• APC CID protects β-catenin from USP7-mediated deubiquitination
• *APC* lacking CID exposes β-catenin to USP7 for deubiquitination
• USP7 depletion inhibits Wnt in *APC* mutant CRC by restoring β-catenin ubiquitination
• USP7 inactivation suppresses xenograft tumor growth and is tumor specific

## Graphical Abstract

**Figure:**
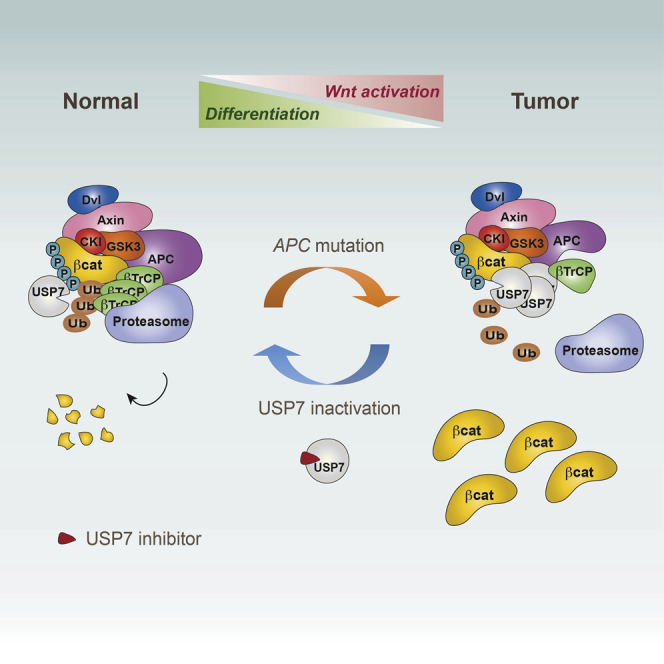

## Teaser

Novellasdemunt et al. find that an *APC*-truncating mutation activates Wnt signaling by promoting USP7-mediated β-catenin deubiquitination. USP7 inhibition significantly suppresses tumor growth of *APC*-mutated colon cancer cells but does not affect cells with wild-type APC, suggesting that it can be used as a tumor-specific drug target.

## Introduction

The canonical Wnt/β-catenin signaling pathway is evolutionarily conserved and plays crucial roles in many biological processes, such as stem cell maintenance and cell fate decision (Clevers and Nusse, 2012). The key downstream modulator of the pathway, β-catenin, is tightly regulated by phosphorylation and ubiquitination-mediated degradation in the cytoplasmic β-catenin destruction complex. The core of the destruction complex consists of the adenomatous polyposis coli (APC), AXIN, glycogen synthase kinase 3 (GSK3), and casein kinase 1 (CK1). β-catenin is first phosphorylated by CK1 and GSK3, followed by recruiting of the E3 ubiquitin ligase β-TrCP to the destruction complex for ubiquitination and subsequent proteasomal degradation (Aberle et al., 1997, Kitagawa et al., 1999, Liu et al., 2002).

Aberrant Wnt activation has been associated with many human cancer types, especially colorectal cancer (CRC) (Clevers and Nusse, 2012, MacDonald et al., 2009, Vogelstein and Kinzler, 2004). Mutations in Wnt pathway components are frequently observed in CRC (Novellasdemunt et al., 2015). The *APC* truncating mutation is the hallmark of the vast majority of human CRCs. Its loss initiates adenoma formation through constitutive Wnt activation (The Cancer Genome Atlas Network, 2012, Nagase and Nakamura, 1993). Most *APC* somatic mutations occur in the “mutation cluster region” (MCR) between codons 1,286 and 1,513 (Polakis, 1995, Vogelstein and Kinzler, 2004). Region-specific *APC* mutations have been associated with distinct β-catenin transcriptional activity and tumor susceptibility (Gaspar and Fodde, 2004). Different functional domains have been described in the central region of the APC protein, including the β-catenin-binding 15- and 20-amino-acid repeats and the Axin-binding Ser-Ala-Met-Pro motif (SAMP) repeats that are crucial for regulating β-catenin level. We have previously shown that APC truncation activates Wnt/β-catenin signaling through abrogation of β-catenin ubiquitination within the destruction complex in CRCs, while assembly of the complex is not affected (Li et al., 2012). However, the molecular mechanism of how APC truncation inhibits β-catenin ubiquitination remains elusive. Several studies have identified yet another highly conserved regulatory domain in APC, the β-catenin inhibitory domain (CID), which is located between the second and the third 20-amino-acid repeats (Kohler et al., 2009, Roberts et al., 2011). The CID is believed to exert an essential role in downregulating β-catenin levels and Wnt transcriptional activity. Importantly, the CID locates right at the MCR that is lost in most human CRCs, suggesting a functional and clinical significance. Despite the crucial role of the CID in Wnt/β-catenin signaling regulation, little is known about the underlying mechanism. APC CID has been shown to interact with α-catenin to promote β-catenin ubiquitination by stabilizing the association with APC as well as to repress β-catenin/T cell factor (TCF) transcription in the nucleus in wild-type APC cells (Choi et al., 2013). A more recent study proposed another speculative model: GSK3-mediated phosphorylation around the CID region promotes a conformational change in APC protein that allows the transfer of phospho-β-catenin to the E3 ligase (Pronobis et al., 2015).

Protein ubiquitination-mediated degradation is a reversible process that is tightly regulated by E3 ubiquitin ligases and deubiquitinating enzymes (DUBs). The role of DUBs in regulating β-catenin ubiquitination is not well defined, particularly in the context of CRC. Here, we sought to investigate the mechanistic role of the *APC* mutation in β-catenin ubiquitination and Wnt activation. We find that the DUB enzyme USP7 is crucial in sustaining pathological, but not physiological, Wnt activation in APC-truncated CRCs by mediating β-catenin deubiquitination.

## Results

### CID Is the Critical Domain for Regulating Wnt Signaling and β-Catenin Ubiquitination

**Figure 1:**
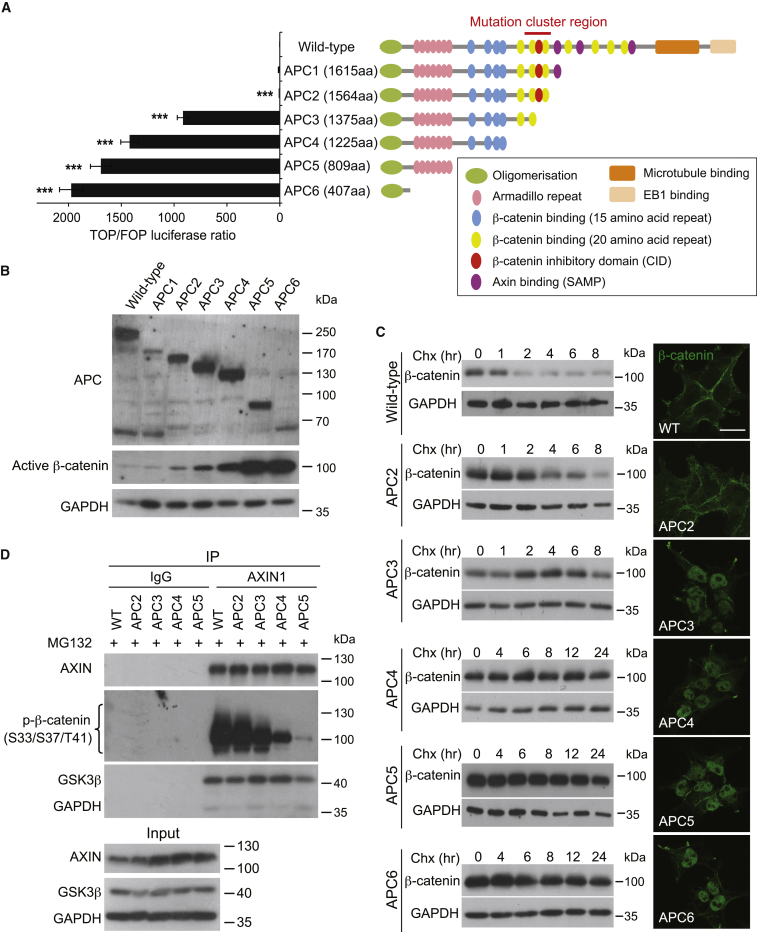
CID Is the Threshold for the Pathological Level of Wnt Activation (A) Schematic representations of human WT APC protein and the corresponding truncating mutants generated by the CRISPR-Cas9 technique. Relative TOP/FOP luciferase activities were measured in different lines. Error bars represent ± SE from at least three independent experiments (^∗∗∗^p < 0.001). (B) Cell lysates of HEK293T WT and APC truncated cell lines were analyzed by western blot using the indicated antibodies. GAPDH protein levels were used as loading control. (C) Cells were treated with cycloheximide (Chx) (50 μg/mL) and collected at different time points as indicated. Stimulated lysates were subjected to western blot analysis using the indicated antibodies. Immunostaining of β-catenin (green) was performed in the indicated cell lines using phospho-insensitive β-catenin antibody (BD Biosciences). Scale bar, 20 μm. (D) Different cell lysates were immunoprecipitated with AXIN1 antibody followed by western blot analysis using the indicated antibodies. IgG, immunoglobulin G. See also Figure S1.

To better understand the role of APC truncation in CRC, we first generated various isogenic lines of *APC* truncating mutations endogenously in wild-type (WT) HEK293T cells with intact Wnt signaling cascade using the CRISPR/Cas9 genome editing technique. Different APC truncating deletions (APC1–6) were generated by targeting the central region of the APC protein sequentially, as shown in Figure 1A (Table S1). A TOPFlash luciferase assay revealed a dramatic upregulation in Wnt/TCF transcription in the APC3 mutant when CID is lost (Figures 1A and S1A). A gradual increase in Wnt signaling was observed upon further truncation (compare APC3–6 in Figure 1A) and confirmed by immunoblotting of APC and active (non-phosphorylated) β-catenin in these mutants (Figures 1B and S1B). Wnt activation of these APC mutants was further analyzed by qRT-PCR of the endogenous Wnt target genes *AXIN2* (Lustig et al., 2002) and *CCND1* (Tetsu and McCormick, 1999) (Figure S1C). Consistently, significant upregulation of these Wnt target genes was observed in APC3–6 mutants. *CTNNB1* mRNA was unchanged among the mutants, demonstrating that accumulation of β-catenin protein occurs post-transcriptionally. Additionally, we did not observe any changes of *APC* transcription, except in APC6 where *APC* mRNA was reduced drastically. This could be due to the instability of the short APC transcript, as previously reported (Dihlmann et al., 1999). We further performed endogenous AXIN1 immunoprecipitation (IP) in our CRISPR-targeted APC mutant cells and confirmed the binding of these truncated APC proteins to the destruction complex (Figure S1D). This is consistent with our previous findings that APC truncation does not cause dissociation of the destruction complex (Li et al., 2012).

Next, we examined the stability of β-catenin in these truncated-APC cells. Significant β-catenin protein degradation was detected at around the 2-hr time point after cycloheximine (Chx) treatment in WT cells, while β-catenin was stabilized in all CID-deleted mutants APC3–6, again with a gradual difference between the various mutants (Figure 1C). Immunofluorescent staining further illustrated the nuclear accumulation of the stabilized β-catenin in these mutants (Figure 1C). Of note, the CID-containing APC2 mutant showed moderate upregulation of Wnt activity, yet without detectable nuclear accumulation of β-catenin.

We have previously shown that *APC* mutations in CRC cells abrogate Axin-bound β-catenin ubiquitination (Li et al., 2012). Here, we examined the β-catenin ubiquitination state by immunoprecipitating the endogenous destruction complex in our CRISPR-targeted APC mutants using AXIN1-specific antibody. Cells were pre-treated with MG132 for 4 hr, followed by endogenous Axin1 IP-coupled western blot analysis. This allows us to visualize the ubiquitinated forms of phosphorylated β-catenin in the Axin pull-down by mobility shift. Consistent with our previous data, suppression of β-catenin ubiquitination was observed in the APC mutants, as revealed by immunoblotting of phospho-β-catenin (S33/S37/T41) antibody (Figure 1D) and phospho-insensitive total β-catenin antibody (Figure S1E). Specifically, we observed a reduction of β-catenin ubiquitination (mobility shift) upon the loss of CID (APC3), with almost-complete abrogation of ubiquitination in the APC4 and APC5 mutants (Figure 1D). Suppression of β-catenin ubiquitination upon APC truncation was further confirmed by co-expressing Myc-Ubiquitin and FLAG-β-catenin, followed by sequential double IP in WT and APC4 cells (Figure S1F). Together, the data support the notion that CID is the pivotal domain of APC to inhibit Wnt signaling by regulating β-catenin ubiquitination.

A previous study proposed an alternative mechanism where *APC* mutation exposes the N-terminal serine/threonine residues of β-catenin to phosphatase for dephosphorylation (Su et al., 2008). To determine whether β-catenin phosphorylation is affected upon a single *APC* truncating mutation around the MCR, we examined GSK3-mediated (S33/S37/T41) and CK1-mediated (S45) β-catenin phosphorylation statuses in WT versus APC2-5 cells in the absence of MG132. In contrast to the previous finding (Su et al., 2008), our data showed robust accumulation of phosphorylated forms of β-catenin in APC3 and APC4 cells upon CID deletion, while phosphorylation was inhibited in APC5 cells (Figure S1G). The result suggests that β-catenin phosphorylation is only affected when all β-catenin binding motifs are lost in the APC protein (APC5), while phosphorylation is still intact in the CID-loss *APC* mutations at the MCR (APC3 and APC4). Our APC CRISPR mutant isogenic cells provide unique tools for direct quantitation of endogenous β-catenin phosphorylation for the first time in a single *APC* mutation event.

### Reciprocal Binding of β-TrCP and USP7 to the β-Catenin-Destruction Complex upon APC Truncation

**Figure 2:**
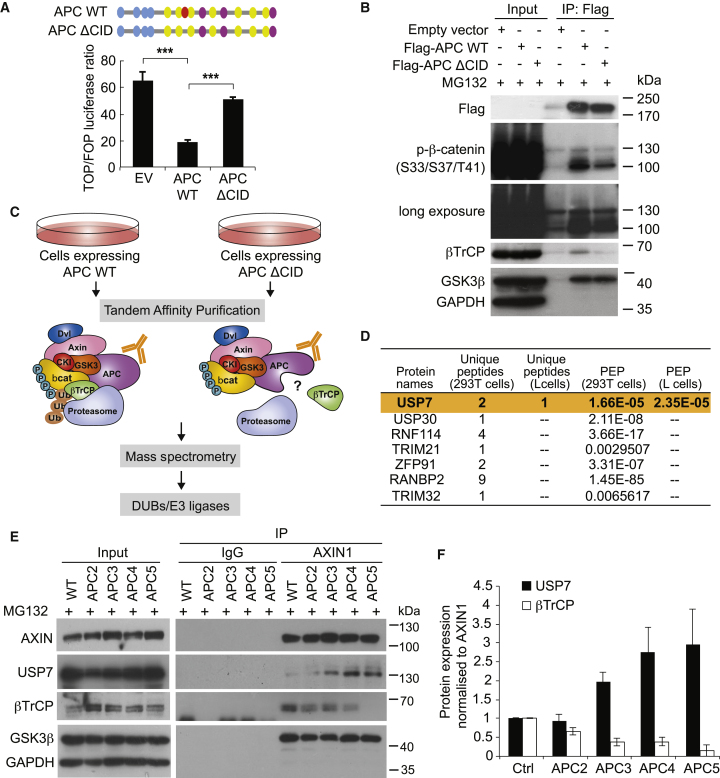
Reciprocal Binding of β-TrCP and USP7 to the β-Catenin-Destruction Complex in APC-Truncated Cells (A) Schematic representation of the central region (1,265–2,060 aas) of APC (APC WT) and the CID-deleted mutant (APC ΔCID). Relative TOP/FOP activities of the APC truncated cell line SW480 transfected with empty vector (EV), APC WT, or APC ΔCID plasmids. Error bars represent SE from at least three independent experiments (^∗∗∗^p < 0.001). (B) HEK293T cells were transfected with EV, FLAG-tagged APC WT, or APC ΔCID. Lysates were subjected to anti-FLAG IP followed by western blotting using the indicated antibodies. (C) Schematic representation of the experimental procedure used for mass spectrometry analysis. (D) DUBs and E3 ligases identified from mass spectrometry in HEK293T and L cells that showed differential bindings to WT versus ΔCID complex. PEP, posterior error probability (p value for protein identification). (E) Cell lysates were immunoprecipitated with AXIN1 antibody followed by western blotting using the indicated antibodies. (F) Quantitation of USP7 and β-TrCP protein binding normalized to AXIN1 protein pulled down in (E). See also Figure S2.

We then asked how β-catenin ubiquitination is inhibited when APC is truncated. We hypothesized that CID regulates β-catenin ubiquitination by interacting with unknown ubiquitin-regulatory proteins. To identify novel CID-bound ubiquitin-regulatory proteins, we generated APC WT and CID-deleted (ΔCID) expression constructs. To test their Wnt inhibitory roles, we expressed the constructs in the *APC* mutant SW480 CRC cells (amino acid [aa] 1338) and measured the Wnt transcriptional activity. TopFlash luciferase assay showed that expression of APC ΔCID failed to suppress Wnt activation in SW480 cells as compared to APC WT (Figure 2A). IP-coupled western blot analysis further demonstrated that β-catenin ubiquitination was abolished in APC ΔCID-expressing cells as revealed by mobility shift (Figures 2B and S2A).

To examine the compositional differences of the β-catenin destruction complex upon the loss of CID, WT and ΔCID APCs were individually expressed in human HEK293T and mouse L cells for tandem affinity purification (TAP) followed by mass spectrometry (MS) analysis (Figure 2C). Intensity-based absolute quantification (iBAQ) was used for label-free quantification. Destruction complex components such as APC, AXIN1, β-catenin, and casein kinases were readily detected in the IP complexes. Protein candidates with differential interaction between APC WT versus ΔCID in either cell line were shortlisted. To identify novel ubiquitin-regulatory proteins, we further shortlisted interactors that are either E3-ubiquitin ligases or DUBs. This resulted in seven protein candidates, among which only the DUB enzyme USP7 (ubiquitin-specific peptidase 7) was detected exclusively in the APC ΔCID complex in both cell lines (Figures 2D and S2B).

Consistent with our MS data, we observed a significant increased binding of USP7 to the destruction complex in the CID-deleted APC mutants (APC3–5) as compared to the WT and the CID-containing APC2 cells (Figures 2E and 2F). Interestingly, an inverse interaction of the E3 ligase β-TrCP to the destruction complex was observed, where binding was significantly decreased in APC3–5 cells. We further confirmed such mutually exclusive presence of USP7 and β-TrCP in an independent experiment with different APC deletion constructs with or without CID (Figures S2C and S2D). Of note, dissociation of β-TrCP to the destruction complex was robust but not complete in the CID-deleted APC mutants (Figures 2B, 2E, S2A, and S2D).

Based on our current data, we speculate that APC CID may, indeed, function to protect β-catenin from binding to the DUB enzyme USP7 in the destruction complex. Deletion of CID will then expose β-catenin to USP7 for deubiquitination, leading to subsequent β-catenin accumulation and aberrant Wnt activation. To test this hypothesis, we co-expressed FLAG-tagged β-catenin and MYC-tagged USP7 in APC4 cells with or without additional CID expression. β-catenin was pulled down using FLAG antibody, and the binding of USP7 in the presence or absence of CID was examined. Consistent with our hypothesis, expression of CID, indeed, suppressed the binding of USP7 to β-catenin (Figure S2E). Together, our data suggest that the CID-loss *APC* mutation promotes β-catenin deubiquitination by (1) reduced binding of the E3 ligase β-TrCP from the destruction complex and (2) exposing β-catenin to the DUB enzyme USP7. This led us to hypothesize the direct interaction between USP7 and β-catenin proteins.

### USP7 Interacts with the N Terminus of β-Catenin Directly to Mediate β-Catenin Deubiquitination

**Figure 3:**
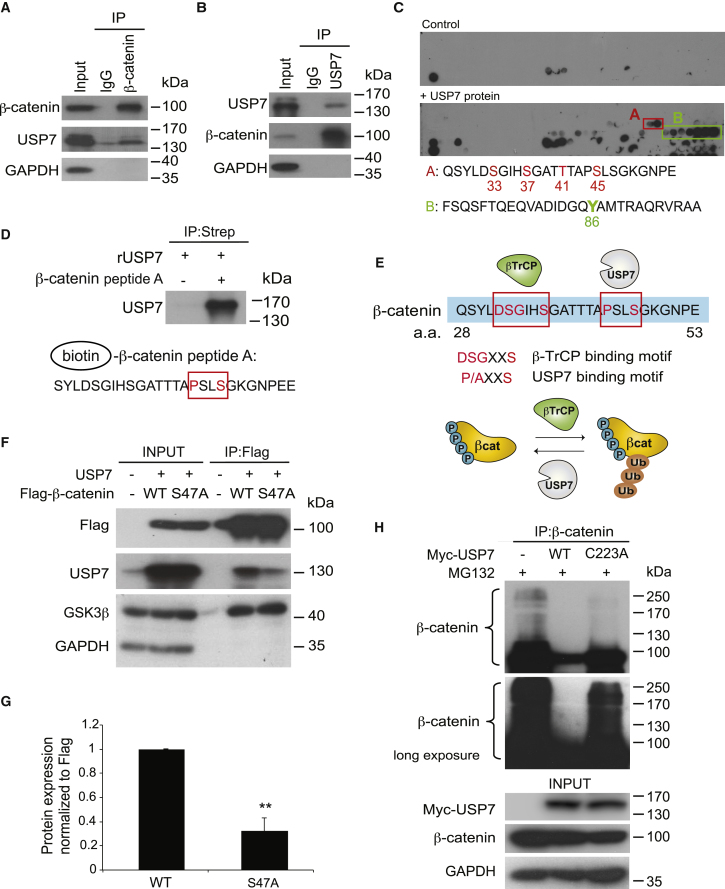
USP7 Directly Interacts with the N Terminus of β-Catenin to Mediate β-Catenin Ubiquitination (A and B) IP of endogenous β-catenin (A) or USP7 (B) in HEK293T cells followed by western blotting using the indicated antibodies. (C) A β-catenin 26-mer peptide array was probed with vehicle control or USP7 recombinant protein. Two USP7-specific binding regions were identified. Reported modification sites were indicated. (D) Biotinylated β-catenin peptide A, including the putative USP7 binding motif (highlighted in the red box), was incubated with recombinant USP7 protein (rUSP7) followed by streptavidin-pull-down assays. (E) Top: presence of β-TrCP and USP7 binding motif in the β-catenin peptide sequence aas 28–53. Conserved sequences are highlighted in red. Bottom: schematic representation of the regulation of β-catenin ubiquitination by β-TrCP and USP7. Phosphorylation sites indicated represent GSK3-mediated (Ser33, Ser37, and Thr41) and CK1-mediated Ser45 phosphorylation. (F) USP7 showed reduced binding to the β-catenin S47A mutant IP complex compared to the WT. (G) Quantitation of the amount of USP7 in the FLAG-β-catenin complex after transfecting β-catenin WT and comparing with β-catenin S47A. Error bars represent SD from three independent experiments. ^∗^p < 0.05. (H) Empty vector, MYC-USP7 WT, or MYC-USP7-C223A plasmids were transfected followed by endogenous β-catenin IP and immunoblotting using the indicated antibodies. See also Figure S3.

Next, we characterized whether β-catenin is the substrate of USP7 for deubiquitination. We first confirmed the interaction between USP7 and β-catenin using endogenous IP in HEK293T cells (Figures 3A and 3B). The binding of USP7 to the Axin1 complex was significantly increased in APC4 mutant compared to WT HEK293T cells (Figure S3A). A previous study suggested that the interaction between USP7 and β-catenin was dependent on another E3 ligase RNF220 (Ma et al., 2014). To validate whether the binding of USP7 to the destruction complex requires RNF220, we performed endogenous IP in both HEK293T and *APC* mutated (APC4) cells using Axin1-specific antibody. In contrast to the reported data, we were unable to detect RNF220 in the endogenous Axin destruction complex (Figure S3A). To further confirm that the USP7 interaction is RNF220 independent, we CRISPR-targeted RNF220 in APC4 cells and repeated endogenous Axin1 IP (Figure S3B). USP7 was detected in the Axin1-pull-down complex in both RNF220-proficient and -deficient cells (Figure S3C). Our results imply that the increased binding of USP7 to the destruction complex upon *APC* mutation is RNF220 independent.

To map the USP7 binding site on β-catenin, we generated a β-catenin 26-mer overlapping peptide array with one residue offset. The array was then probed with USP7 recombinant protein or control to identify specific binding signal. Two potential USP7 binding sites were detected on the N terminus of β-catenin (Figure 3C). To validate the USP7 binding, we synthesized the two β-catenin peptides identified from the array with N-terminal biotin labeling for IP analysis. Our results confirmed the direct interaction of USP7 to the two identified β-catenin peptide regions (Figures 3D and S3D). Interestingly, the first peptide region (aas 28–53) covers the GSK3 (Ser33, Ser37, and Thr41) and CK1 (Ser45) phosphorylation sites, while the second region (aas 70–97) covers another reported modification site, Tyr86 (Tominaga et al., 2008). Motif analysis confirmed a β-TrCP binding motif (aas 32–37; DSGIHS) and revealed a USP7 binding motif (aas 44–47; PSLS) in the first detected peptide (Figure 3E) (Sheng et al., 2006). To validate whether “PSLS” is the USP7 binding motif on β-catenin, we mutated the consensus serine residue (Ser47) on β-catenin (i.e., “PSLS” to “PSLA”) and examined the interaction with USP7. IP-coupled western blotting demonstrated a significantly weaker interaction between USP7 and β-catenin S47A mutant protein as compared to the wild-type β-catenin (Figures 3F and 3G). We further generated the β-catenin Y86A mutant to test the second putative USP7 binding region. Pull-down of β-catenin Y86A also showed a moderate decrease in binding to the USP7 protein (Figures S3E and S3F), although the decrease was not as significant as in the S47A mutant. Of note, the close proximity of both USP7 and the β-TrCP binding motif on the N terminus of β-catenin suggests a potential competition of substrate between the two enzymes.

Finally, we tested whether β-catenin is the direct substrate of USP7 by generating a catalytic inactive mutant of USP7 C223A (Hu et al., 2002). Expression of WT USP7 robustly suppressed β-catenin ubiquitination, as demonstrated by mobility shift, while the C223A mutant expression failed to execute its function (Figures 3H and S3G). An in vitro deubiquitination assay further confirmed that β-catenin is the direct substrate of USP7 (Figure S3H). Together, our data imply that USP7 interacts with the N terminus of β-catenin directly to mediate β-catenin deubiquitination when *APC* is mutated.

### USP7 Depletion in APC-Truncated CRC Cells Suppresses Aberrant Wnt Activation by Restoring β-Catenin Ubiquitination

**Figure 4:**
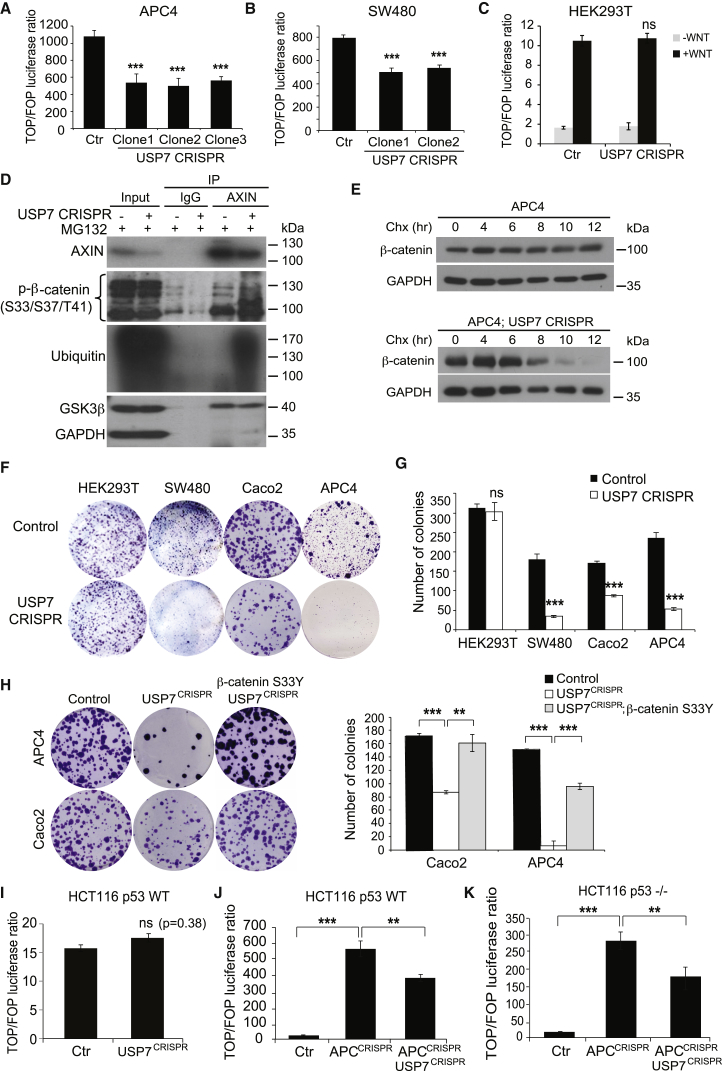
USP7 Depletion in APC-Truncated CRC Suppresses Wnt Activation by Restoring β-Catenin Ubiquitination (A–C) Relative TOP/FOP activities of the APC4 (A), SW480 (B), and HEK293T (C) cells with control or USP7 CRISPR targeting. (D) AXIN1 complexes were immunoprecipitated in APC4 cells with or without USP7 targeting followed by western blotting using the indicated antibodies. (E) Cells were treated with cycloheximide (Chx) (50 μg/μL), and lysates were collected at different time points as indicated for immunoblotting of ABC (active β-catenin) and control GAPDH. (F) Colony formation assay in parental HEK293T, SW480, Caco2, and APC4 cells and the corresponding USP7 CRISPR-deleted cells. (G) Quantitation of number of colonies in (F). Experiments were performed in triplicates. (H) Colony formation assay of APC4 and Caco2 cells upon transient transfection of USP7 CRISPR and/or β-catenin S33Y mutant plasmids. The corresponding quantitation is indicated on the right. (I–K) Relative TOP/FOP activities of the HCT116 p53 WT (I and J) and HCT116 p53^−/−^ (K) cells with indicated CRISPR targeting. Error bars represent SE from at least three independent experiments (^∗∗^p < 0.01; ^∗∗∗^p < 0.001; ns, not significant). See also Figure S4.

Our current data support a model in which APC truncation activates Wnt signaling by exposing the destruction-complex-bound β-catenin to the DUB enzyme USP7 for deubiquitination. To test whether this process is reversible, we depleted USP7 in the *APC* mutant cell line (APC4) using CRISPR/Cas9 gene targeting. Wnt activation of APC4 cells was significantly suppressed upon *USP7* mutation (Figures 4A and S4A–S4C). Interestingly, we were not able to obtain stable knockout (KO) of USP7 efficiently in APC4 cells. Transient targeting of USP7 in APC4 cells immediately after transfection significantly suppressed Wnt transcriptional activity, while such suppression was gradually lost upon colony picking (Figure S4B). We reasoned that USP7 depletion in *APC* mutant cells might have a growth disadvantage and may, therefore, be rapidly replaced by USP7 WT cells upon passaging. Indeed, the USP7 targeted cells grew much more slowly than the non-targeted cells. USP7 has been previously described in the p53/Mdm2 pathway to regulate p53-dependent apoptosis (Li et al., 2002, Meulmeester et al., 2005). To prove that the USP7-mediated Wnt activation is p53 independent, we further targeted USP7 in the *APC*-mutated CRC cell line SW480 carrying the *p53* mutation. Similar to the APC4 cells, we were not able to obtain complete USP7 KO in SW480 cells (Figure S4D). Transient targeting of USP7 in SW480 cells showed robust suppression of USP7 protein and Wnt activation, while the USP7-depleted cells were rapidly lost upon passaging and colony picking (Figures S4E and S4F). Despite the incomplete USP7 depletion, a significant suppression in Wnt signaling was observed usinig TopFlash luciferase and Wnt target gene transcription (Figures 4B and S4G). The results showed that USP7-mediated Wnt activation in CRC is a reversible process. The rapid loss of USP7-depleted cells suggests that USP7 might be essential for the survival of *APC* mutant cells.

To test whether USP7 is also important in physiological Wnt activation, we CRISPR targeted USP7 in WT HEK293T cells with intact APC and measured the TopFlash luciferase activity upon Wnt3A induction. In contrast to APC4 and SW480 cells, complete KO of USP7 could be generated easily in the WT HEK293T cells (Figure S4H; Table S2). Surprisingly, we did not observe any changes in Wnt activation, despite the complete USP7 KO (Figure 4C). Our data suggest that USP7 is essential to sustain pathological Wnt activation in *APC* mutants but not physiological Wnt activation in normal cells.

We next tested whether USP7 depletion in *APC*-mutated cells was able to rescue β-catenin ubiquitination. Indeed, CRISPR targeting of USP7 in the CID-deleted APC4 cells readily restored β-catenin ubiquitination in the destruction complex (Figures 4D and S4I). To further demonstrate that the ubiquitination event is β-catenin specific, we co-transfected His-tagged ubiquitin and FLAG-tagged β-catenin in APC4 cells with or without USP7 depletion for double IP. Western blot analysis of the His pull-down samples showed significantly more ubiquitinated β-catenin in APC4 cells after USP7 depletion, as shown by the shift of higher molecular weight forms of β-catenin (Figure S4J). Consistent with our IP and TopFlash luciferase data, β-catenin protein degradation was significantly accelerated when USP7 was depleted in APC4 cells (Figures 4E and S4K). We concluded that USP7 is essential in mediating β-catenin deubiquitination and aberrant Wnt activation upon APC truncation.

Next, we examined whether USP7 deletion affects cell proliferation and survival. Clonogenic assays were performed in Caco2, SW480, APC4, and HEK293T cells with or without USP7 CRISPR targeting. Depletion of USP7 in Caco2, SW480, and APC4 cells significantly suppressed colony formation in comparison to the parental cells (Figures 4F and 4G). In contrast, USP7 KO in WT HEK293T cells did not show any effect on cell growth. To further validate that the growth suppression of USP7 depletion in APC mutants is, indeed, due to Wnt/β-catenin signaling, we introduced a stabilized form of β-catenin (Ser33Y mutant) in the APC4 and Caco2 cells upon USP7 CRISPR targeting. β-Catenin (Ser33Y) expression significantly rescued the number of colonies formed as well as the Wnt transcriptional activity mediated by USP7 CRISPR mutation in both cells (Figures 4H and S4L), suggesting that the growth defect upon USP7 loss is due to modulation of Wnt signaling. Our data support the notion that USP7 plays a crucial role in Wnt signal activation and cell survival, specifically in APC truncated cells.

To further test whether USP7 also regulates Wnt signaling in non-*APC*-mutated CRC, we depleted USP7 in another CRC cell line, HCT116, carrying WT APC and heterozygous β-catenin Ser45 deletion (Figure S4M). Similar to the WT HEK293T cells, CRISPR targeting of USP7 in HCT116 cells did not suppress Wnt activation (Figure 4I). We further interrogated the cell line by introducing an additional *APC* mutation using CRISPR targeting (HCT116 APC^CRISPR^). Unlike the parental cell line, USP7 depletion in HCT116 APC^CRISPR^ cells showed significant Wnt suppression (Figure 4J). We performed similar experiments in HCT116-p53 null cells and observed similar results (Figures 4K, S4N, and S4O). Of note, we were not able to obtain complete APC KO in the USP7-depleted HCT116 cells for the same growth disadvantage reason as that observed in APC4 and SW480 cells, confirming that USP7 is essential for cell survival in *APC*-mutated cells. Collectively, we concluded that the USP7-mediated β-catenin deubiquitination and Wnt activation are specific to CRCs carrying *APC* mutations and are p53 independent.

### Targeting USP7 with Small-Molecule Inhibitors Suppresses Wnt Activation in CRCs Carrying CID-Deleted *APC* Mutations

**Figure 5:**
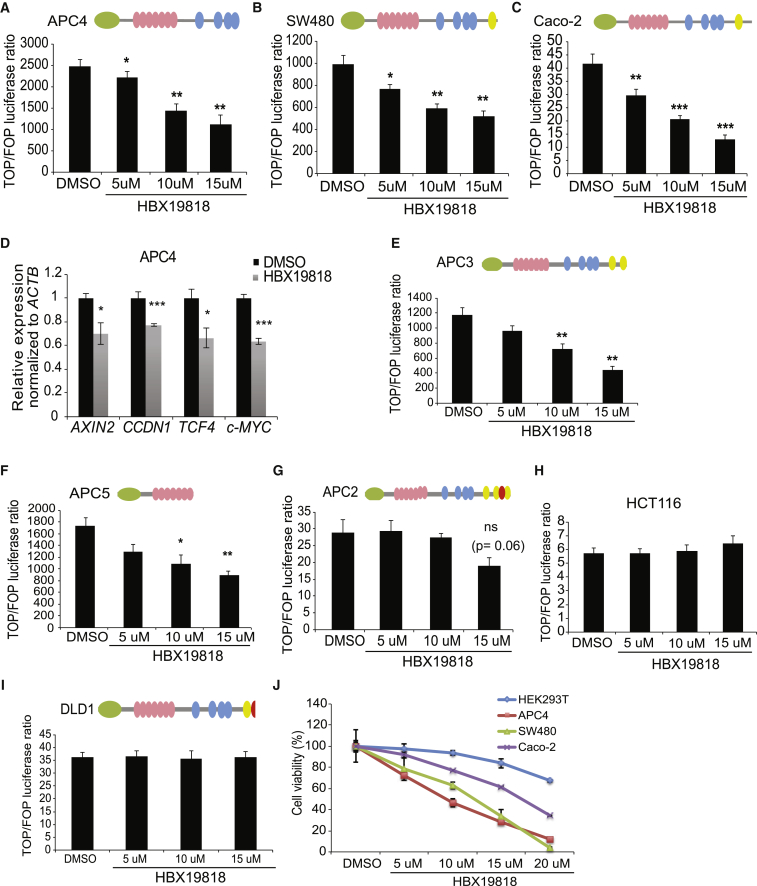
USP7 Inhibitor Treatment Suppresses Wnt Activation in CID-Deleted *APC* Mutant CRCs (A–C) Relative TOP/FOP activities of APC4 (A), SW480 (B), and Caco-2 (C) cells treated with HBX19818 at the indicated concentrations or DMSO as control. (D) mRNA expression of the indicated Wnt target genes was analyzed by qRT-PCR in APC4 cells. Data are presented as fold change normalized to *β-actin* control in triplicate and are representative of at least three independent experiments. (E–I) Relative TOP/FOP activities of APC3 (E), APC5 (F), APC2 (G), HCT116 (H), and DLD1 (I) cells treated with DMSO or HBX19818 at the indicated concentrations. (J) MTT assay in the indicated cell lines treated with DMSO or HBX19818 at the indicated concentrations for 24 hr. Error bars represent ± SE from at least three independent experiments (^∗^p < 0.05; ^∗∗^p < 0.01; ^∗∗∗^p < 0.001; ns, not significant). See also Figure S5.

Our current data indicate that USP7 is a potential tumor-specific drug target for *APC*-mutated CRCs by altering the Wnt signaling pathway. Several USP7 inhibitors have been previously developed to target the p53/Mdm2 pathway, while their roles in the Wnt signaling pathway have not been addressed (Fan et al., 2013, Reverdy et al., 2012). Here, we examined the effect of the USP7-specific inhibitor HBX19818 on Wnt signaling in CRC cells (Reverdy et al., 2012). Treatment of the CRISPR-targeted APC4 cells with HBX19818 showed a marked reduction in Wnt activation (Figure 5A). Similar results were obtained in CRC cells carrying CID-deleted *APC* mutations (SW480 and Caco-2) (Figures 5B and 5C). Significant suppression of Wnt target gene transcription was also confirmed in APC4 after HBX19818 treatment (Figure 5D).

To validate whether the function of USP7 is specific to CID loss, we further examined the effect of HBX19818 on the different CRISPR-targeted isogenic APC mutants. Consistent with our hypothesis, Wnt signaling was significantly suppressed in the CID-deleted APC3 and APC5 cells but not in the CID-containing APC2 cells upon inhibitor treatment (Figures 5E–5G). Similarly, CRC cells carrying WT APC (HCT116) or CID partly containing *APC* mutation (DLD1) and WT HEK293T treated with HBX19818 did not suppress Wnt activation (Figures 5H, 5I, and S5A). A 3-(4,5-dimethylthiazol-2-yl)-2,5-diphenyltetrazolium bromidefor (MTT) assay further revealed that HBX19818-treated cells showed significantly reduced cell viability in the APC-truncated cells when compared to HEK293T cells (Figure 5J).

To validate the Wnt suppression effect, we further tested another reported USP7-specific inhibitor, P22077 (Fan et al., 2013). Similar to HBX19818, P22077 treatment demonstrated a dose-dependent Wnt suppression in the APC-truncated APC4 and SW480 cells but not in the WT APC HEK293T or HCT116 cells (Figures S5B–S5E). Significant suppression of Wnt target gene transcription was also confirmed in APC4 cells after P22077 treatment (Figure S5F). An MTT assay further confirmed that P22077-treated cells significantly inhibited the cell viability of the APC-truncated cells as compared to HEK293T cells at the equivalent dose (Figure S5G).

In summary, USP7 inhibition by CRISPR targeting or treatment with two different small molecules consistently suppresses aberrant Wnt activation in CID-loss APC mutant cells. Our data highlight the potential of USP7 as a tumor-specific drug target for CRCs carrying CID-deleted *APC* mutations.

### Inactivation of Usp7 in *Apc*-Mutated Intestinal Organoids Promotes Differentiation and Suppresses Growth

**Figure 6:**
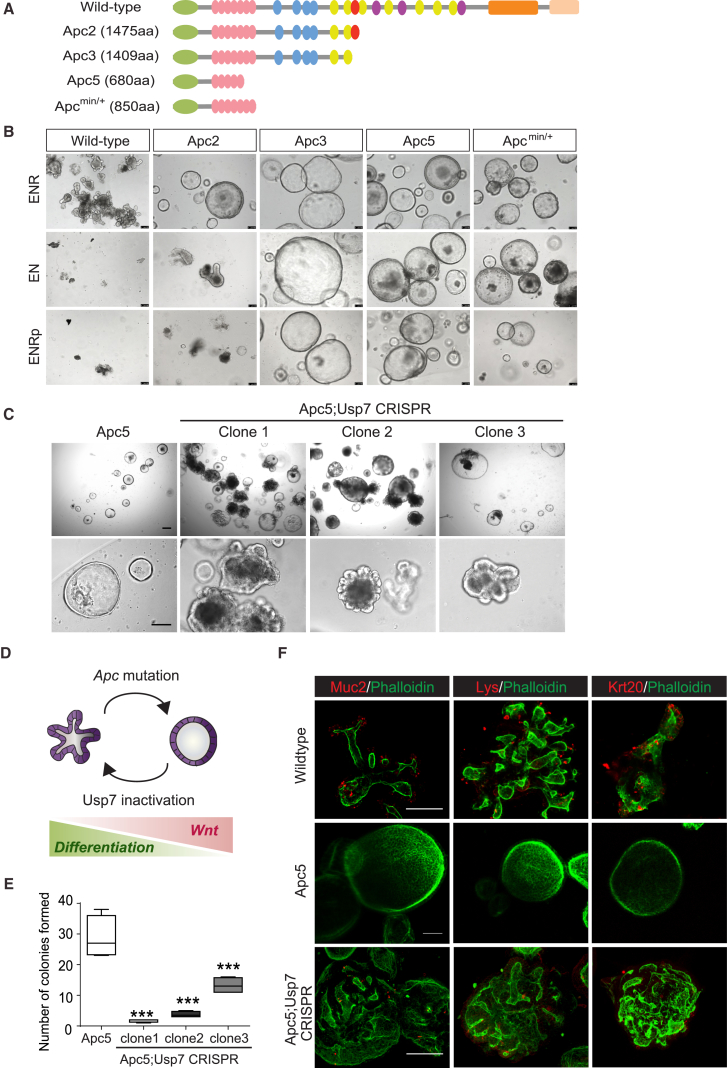
Usp7 Deletion in *Apc*-Mutated Intestinal Organoids Induces Differentiation and Suppresses Growth (A) Schematic representation of the mouse WT Apc protein and the corresponding truncating mutants generated by the CRISPR-Cas9 technique. (B) Morphological changes of organoids cultured in the indicated conditions. E, Egf; N, Noggin, R, R-spondin; p, porcupine inhibitor (IWP2). Scale bars, 100 μm. (C) Representative images of Apc5 organoids with or without *Usp7* CRISPR targeting. Scale bars, 100 μm. (D) Schematic representation of the changes on the morphology of intestinal organoids upon *Apc* loss and *Usp7* inactivation. (E) Clonogenic assay of Apc5 organoids with or without *Usp7* CRISPR targeting after 7 days in culture. Error bars represent SE from at least three independent experiments (^∗∗∗^p < 0.001). Scale bar, 100 μm. (F) Immunofluorescent staining of WT, Apc5, and Apc5;Usp7 CRISPR-targeted organoids using the indicated antibodies. Red signal shows markers of differentiation, including *Mucin2* (Muc2), *Lysozyme* (Lys), and *Keratin20* (Krt20). Scale bars, 100 μm. See also Figure S6.

Next, we studied the functional significance of Usp7 in ex vivo intestinal organoid culture (Sato et al., 2009). We first engineered different APC truncations in organoids using the CRISPR/Cas9 technique and compared their growth upon functional selection (Figure 6A; Table S3). Organoids derived from tumors isolated from Apc^min/+^ mice were used as positive controls (Sato et al., 2011). Normal intestinal organoids that are cultured in a previously described medium (ENR) will form a budding structure with multi-lineage differentiation, while organoids with Apc depletion will turn into tumor-like “spheroids” that are hyperproliferative and extracellular Wnt signal independent and that lack differentiation (Dow et al., 2015, Drost et al., 2015). Similar to the Apc^min/+^ organoids, all three engineered *Apc* mutant organoids showed spheroid morphology (Figure 6B, top panel). We asked whether our engineered *Apc* mutant organoids are dependent on exogenous Wnt signal by R-spondin withdrawal (Schwank et al., 2013) or treatment with porcupine inhibitor (which blocks Wnt ligand secretion) (Chen et al., 2009). Both WT and CID-containing Apc2 organoids died after exogenous Wnt depletion, whereas the CID-deleted Apc3, Apc5, and Apc^min/+^ organoids could be maintained as spheroids in the culture (Figure 6B). Taken together with our CRISPR-engineered cell line data, the results support the notion that CID is the critical threshold in APC for the pathological level of Wnt activation and tumor transformation.

To determine whether Usp7 is essential in maintaining tumor-like spheroid growth in Apc-*mutant* organoids, we deleted Usp7 in Apc5 organoids by CRISPR targeting. Usp7 depletion prominently reversed the spheroid phenotype in Apc5 that showed recovery of organoid sprouting—evidence of normal differentiation (Figures 6C, 6D, and S6A; Table S3). Wnt signaling was also significantly suppressed upon *Usp7* mutation (Figure S6B). Similar to the cell line data, we were not able to maintain Usp7 KO in Apc5 organoids. Indeed, the Usp7 KO-induced sprouting phenotype was rapidly lost upon passaging, suggesting the growth disadvantage of these cells upon Usp7 loss (Figure S6C). Despite the incomplete KO, inhibition of Usp7 in Apc5 organoids resulted in significant growth suppression (Figures 6E, S6D, and S6E) and cellular differentiation (Figures 6F and S6F). Importantly, CRISPR-targeted *Usp7* mutation in WT organoids did not show any significant effect, which is consistent with the tumor-specific role we observed in CRC cell lines (Figures S6G and S6H; Table S3). Together, our data suggest that USP7 can be used as a tumor-specific therapeutic target to suppress tumor growth and induce cellular differentiation via Wnt inactivation.

### USP7 Depletion Significantly Inhibits *APC*-Mutated CRC Tumor Growth In Vivo

**Figure 7:**
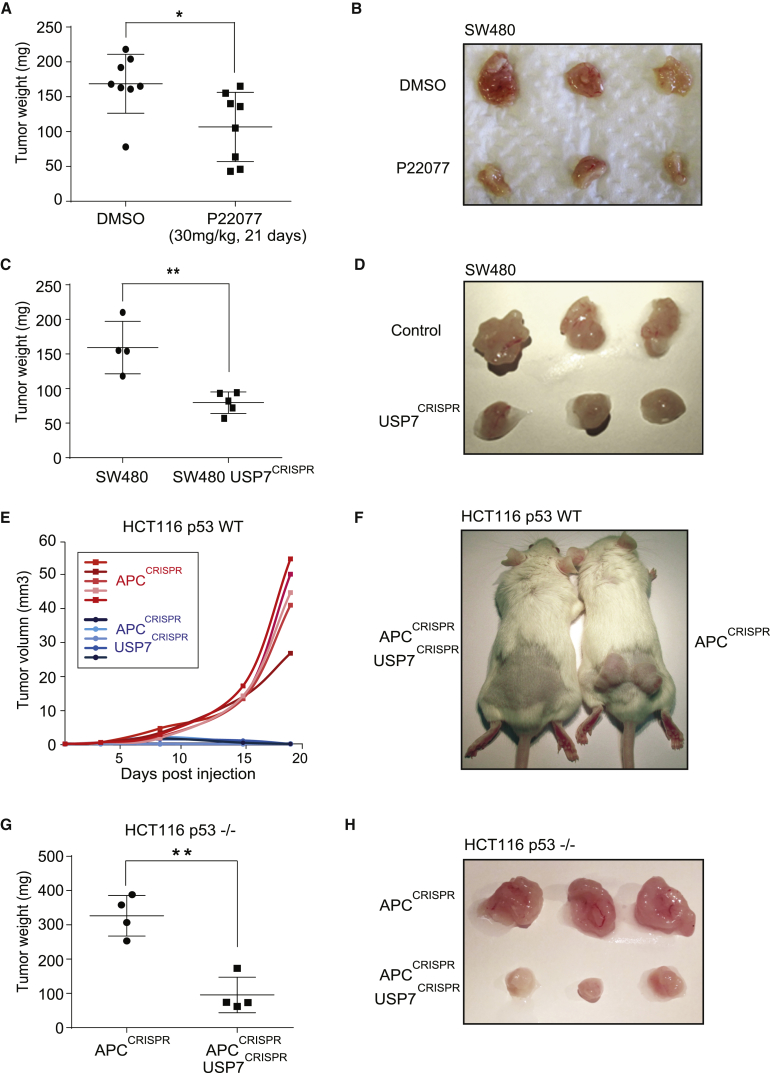
USP7 Inactivation Suppresses *APC*-Mutated Colorectal Cancer Tumor Growth In Vivo (A) SW480-derived tumor weights between DMSO control group and P22077 treatment group (30 mg/kg) at the end of treatment (21 days) (^∗^p < 0.05) (n = 8 per condition). (B) Representative photos of SW480-derived tumors at the end of treatment. (C) Comparison of weights between parental SW480- and SW480 USP7 CRISPR-derived tumors at the end of treatment (25 days) (^∗∗^p < 0.01) (n = 4 and n = 5, respectively). (D) Representative photos of SW480 and SW480 USP7 CRISPR-derived tumors at the end of treatment. (E) Tumor volumes derived from HCT116 APC^CRISPR^ compared to HCT116 APC^CRISPR^ USP7^CRISPR^ throughout the experiment (19 days) (n = 5 per condition). (F) Representative photos of the mice injected with HCT116 APC^CRISPR^ cells compared to HCT116 APC^CRISPR^ USP7^CRISPR^ cells at the end of the experiment. (G) Weight comparison between HCT116 p53^−/−^ APC^CRISPR^- and HCT116 p53^−/−^ APC^CRISPR^ USP7^CRISPR^-derived tumors at the end of the experiment (13 days) (n = 4 per condition; ^∗∗^p < 0.01). (H) Representative photos of the tumors derived from HCT116 p53^−/−^ APC^CRISPR^ and HCT116 p53^−/−^ APC^CRISPR^ USP7^CRISPR^ cells. See also Figure S7.

To confirm the therapeutic role of USP7 in CRC, we further studied the effect of USP7 inhibition in vivo using a xenograft model. Colon cancer SW480 cells were injected subcutaneously into the flank of SCID mice. USP7 inhibitor P22077 or vehicle control (DMSO) was administered at 30 mg/kg daily by intraperitoneal injections (Fan et al., 2013). Treatment of the mice with P22077 significantly suppressed tumor growth compared to vehicle treatment (Figures 7A and 7B). Mice treated with P22077 did not show any detectable health problems or weight loss (Figure S7A). Histological analysis of the intestine from the P22077-treated mice also showed no significant difference compared to the control animals (Figure S7B). In addition, tumor material isolated from the P22077-treated mice showed suppression of Wnt target genes (Figure S7C). The data suggest that USP7 inhibitors can be used for treatment of *APC*-mutated CRC by suppressing pathological Wnt activation.

To validate the tumor-suppressive effect of USP7 inhibitors, we repeated the xenograft experiment by comparing tumor growth between the parental and USP7 CRISPR-targeted SW480 cells (Figure S7D). Consistent with the inhibitor data, USP7 depletion significantly inhibited tumor growth, compared to the parental SW480 tumors (Figures 7C and 7D) with reduced Wnt target gene expression (Figure S7E).

We further tested whether the tumor-suppressive effect is p53 dependent by comparing the effect of Usp7 depletion in our CRISPR-engineered HCT116 cell line carrying an additional *APC* mutation (HCT116 APC^CRISPR^) in both p53 WT and null backgrounds. Strikingly, the HCT116 APC^CRISPR^ USP7^CRISPR^ cells demonstrated remarkable suppression in tumor growth when compared to the HCT116 APC^CRISPR^ cells (Figures 7E and 7F). To confirm that the USP7-mediated tumor suppression is p53 independent, we compared the xenograft tumor growth between HCT116 p53^−/−^ APC^CRISPR^ and HCT116 p53^−/−^ APC^CRISPR^ USP7^CRISPR^ cells. Consistently, USP7 depletion dramatically suppressed tumor growth in the HCT116 p53^−/−^ background (Figures 7G and 7H). In summary, our findings suggest that USP7 is a promising tumor-specific target for treatment of *APC*-mutated CRC by targeting pathological Wnt activation.

## Discussion

The connection between the Wnt signaling pathway and CRC was first identified in the early 1990s. Both somatic and germline mutations of *APC* were discovered in CRC patients in 1991 (Groden et al., 1991, Joslyn et al., 1991, Kinzler et al., 1991, Nishisho et al., 1991), while the interaction between APC and β-catenin was found 2 years after (Rubinfeld et al., 1993, Su et al., 1993). Despite extensive research on Wnt signaling and CRC over the past 20 years, clinically approved drugs targeting Wnt signaling in *APC* mutation cancer do not exist. The major hurdle of therapeutic development of Wnt pathway inhibitors rests in its essential role in adult tissue homeostasis. Drugging Wnt signaling will inevitably cause toxicity to Wnt-dependent normal tissue development such as in the intestine, thus limiting the full antitumor efficacy. Development of tumor-specific Wnt inhibitor would circumvent this challenge.

In this study, we identified USP7 as a tumor-specific target by characterizing the fundamental Wnt-activating mechanism of *APC* mutation. Most previous studies on APC and the Wnt pathway relied heavily on ectopic overexpression of various *APC* mutant proteins in non-physiological doses. None of these experiments can faithfully recapitulate the endogenous molecular changes upon a single, specific *APC* mutation in a mammalian cell. Here, we engineered various endogenous APC truncations in WT isogenic cell lines and intestinal organoids using CRISPR/Cas9 genome editing and revealed the functional significance of the CID domain in APC as the critical threshold for pathological Wnt activation and tumor formation. Our data show that CID-lacking APC truncation results in Wnt pathway activation by facilitating binding of the DUB enzyme USP7—at the cost of binding of the E3 ligase β-TrCP—to the destruction complex for β-catenin deubiquitination. Inactivation of USP7, either by CRISPR mutation or by small-molecule inhibitors, suppresses *APC*-mutated CRC tumor growth and Wnt activation by restoring β-catenin ubiquitination. Importantly, USP7 inhibition does not affect normal cells with WT APC, indicating that USP7 can be used as a tumor-specific drug target for *APC*-mutated CRCs. Additional in vivo experiments using USP7 floxed mice in combination with an *Apc*-mutated tumor model will be crucial to confirm the tumor-suppressive effect upon USP7 depletion in the intestine. Our current data further imply a critical role of APC CID in regulating β-catenin ubiquitination by protecting β-catenin from interacting with the DUB enzyme USP7. Ubiquitination of other proteins in the destruction complex, such as AXIN and APC, has been previously reported (Tran et al., 2013, Zhang et al., 2011). It would be interesting to further examine whether USP7 can target other proteins in the destruction complex apart from β-catenin.

Thus far, only two studies have attempted to examine the role of USP7 in Wnt signaling. The first study showed that USP7 is not involved in Wnt signaling based on short hairpin RNA (shRNA) screening in HEK293T cells (Tauriello and Maurice, 2010). Another group recently proposed, using a small interfering RNA (siRNA) knockdown strategy, that USP7 enhances Wnt signaling through RNF220-dependent β-catenin deubiquitination (Ma et al., 2014), which is inconsistent with our current findings. In fact, our data are consistent with the previous finding that USP7 depletion does not affect physiological Wnt activation. We show that USP7-mediated β-catenin deubiquitination is a tumor-specific event when APC is truncated and that it is RNF220 independent. Our data not only clarify the tumor-specific role of USP7 in Wnt signaling but also highlight the importance of developing physiological experimental models such as CRISPR-engineered mutations for molecular and functional studies. Of note, our current data do not completely exclude the role of USP7 in physiological Wnt regulation. In fact, recent studies have reported the roles of other DUB enzymes such as CYLD, USP47, and USP6 in physiological Wnt regulation (Madan et al., 2016, Shi et al., 2015, Tauriello and Maurice, 2010). It is, therefore, possible that USP7 inhibition alone does not affect physiological Wnt signaling due to redundancy. Further investigation will be needed to delineate the potential unique or redundant roles of these DUB enzymes in physiological and pathological Wnt regulation.

Previous studies have identified other proteins as potential substrates of the DUB enzyme USP7 (Nicholson and Suresh Kumar, 2011). The tumor suppressor gene p53 was first identified as the primary substrate of USP7 for deubiquitination and stabilization (Li et al., 2002). Subsequent studies further described the USP7-mediated deubiquitination of the E3 ligases of p53, Mdm2/HDM2 (the human ortholog of Mdm2), and its homolog HDMX, suggesting a dynamic role of USP7 in regulating the p53/Mdm2 pathway (Li et al., 2004, Meulmeester et al., 2005). To address whether the Wnt-activating role of USP7 is p53 dependent, we tested CRC cells with the *p53* mutation (SW480 and HCT116 p53^−/−^). Our results clearly demonstrated that USP7-mediated β-catenin deubiquitination is p53 independent. We confirmed that targeting USP7 can suppress tumor growth in vivo by inhibiting Wnt activation. To our knowledge, this is the first intervention against USP7 in CRC in vivo in the context of the Wnt signaling pathway.

Given the critical role in regulating both p53/Mdm2 and Wnt/β-catenin signaling, USP7 holds great promise as a therapeutic target for cancer treatment. Our current findings have added significant implications to the development and application of USP7 inhibitors in the clinic. The fact that we were not able to obtain complete USP7 KO in *APC*-mutated CRC cells indicates the essential role of USP7 in cancer cell survival. On the other hand, incomplete KO of USP7 was sufficient to inhibit Wnt signaling and suppress tumor growth. Our data echo a recent study showing that minimal APC restoration is sufficient for tumor suppression (Dow et al., 2015). This further implies a promising safe and efficient therapeutic window of USP7 inhibitors for cancer treatment. Identifying the target population is a critical step for developing a successful drug with high efficacy. Our data suggest that USP7 inhibitors can be used for treatment of CID-deleted *APC* mutated CRCs, as well as for potential preventive therapy for FAP patients carrying germline *APC* mutations, by delaying cancer onset. Since the Wnt-activating role of USP7 is tumor specific and p53 independent, drugs targeting USP7 can potentially be used to treat CRC patients with *APC* mutations, regardless of *p53* status. However, we believe that USP7 inhibitor treatment will be more effective in patients carrying the *APC* mutation and WT p53 due to the synergistic effect of Wnt signal suppression and p53 stabilization. Further studies on the structural interaction between USP7 and β-catenin, as well as the conformational changes of the APC protein upon the CID loss, may aid the development of improved Wnt-specific USP7 inhibitors.

## Experimental Procedures

### Cell Culture, Transfection, and TOPFlash Assay

HEK293T, SW480, DLD1, HCT116, and Caco-2 cells were maintained in DMEM GlutaMAX (GIBCO) supplemented with 5% fetal bovine serum (FBS) (GIBCO) and with 100 U/mL penicillin (GIBCO) and 100 μg/mL streptomycin (GIBCO). All cells were maintained at 37°C in an incubator with 5% CO_2_. Cells were seeded in plates 24 hr before transfection, and plasmids were transfected using polyethylenimine (Polysciences) according to the manufacturer’s instructions. For the TOPFlash luciferase assay, cells in a 48-well plate were transfected with 100 ng of the reporter plasmid TOP or FOP and 10 ng of TK/Renilla for each well. 100 ng of additional plasmid was cotransfected in each well when indicated. After 16 hr of transfection, cells were treated with Wnt3a-conditioned medium (when indicated) or control medium or with USP7 inhibitor treatment or DMSO for an additional 18 hr. The luciferase activity was measured using a luminometer. For transfections for IP, cells were also seeded 24 hr before transfection in a 10- or 15-cm plate, and 7–15 μg plasmid was transfected in each plate dependent on each plasmid. 48 hr after transfection, cells were lysed.

### IP and Immunoblotting

Cells were pre-treated with 10 μM MG132 proteasome inhibitor for 4 hr prior to lysate collection for all IP experiments. Cells were washed and collected with cold PBS and lysed in cold lysis buffer as previously described (Li et al., 2012).

### Animal Procedures

All animal-regulated procedures were carried out according to Project License constraints and Home Office guidelines and regulations.

See the Supplemental Experimental Procedures for additional methods.

## Author Contributions

L.N. and V.S.W.L. designed the experiments and analyzed the data. L.N., V.F., L.C., P.A., A.K., V.E., and A.P.S. conducted the experiments. L.N. and V.S.W.L. wrote the paper.

## Data and Software Availability

Mass spectrometry data reported in this paper have been deposited in the ProteomeXchange Consortium via the PRIDE partner repository with the dataset identifier ProteomeXchange: PXD007947.

## Supplemental Information

Document S1. Supplemental Experimental Procedures, Figures S1–S7, and Tables S1–S3

Document S2. Article plus Supplemental Information
