# Supplementary material for: USP7 Is a Tumor-Specific WNT Activator for APC-Mutated Colorectal Cancer by Mediating β-Catenin Deubiquitination
Source: Cell Rep. 2017 Oct 17;21(3):612–27. doi: 10.1016/j.celrep.2017.09.072 (PMC5656747; doi:10.1016/j.celrep.2017.09.072)
Supplement: Document S1. Supplemental Experimental Procedures, Figures S1–S7, and Tables S1–S3 [file mmc1.pdf]

Cell Reports, Volume *21*

Supplemental Information

USP7 Is a Tumor-Specific WNT Activator for *APC*-Mutated Colorectal Cancer by Mediating β-Catenin Deubiquitination

Laura Novellasdemunt, Valentina Foglizzo, Laura Cuadrado, Pedro Antas, Anna Kucharska, Vesela Encheva, Ambrosius P. Snijders, and Vivian S.W. Li

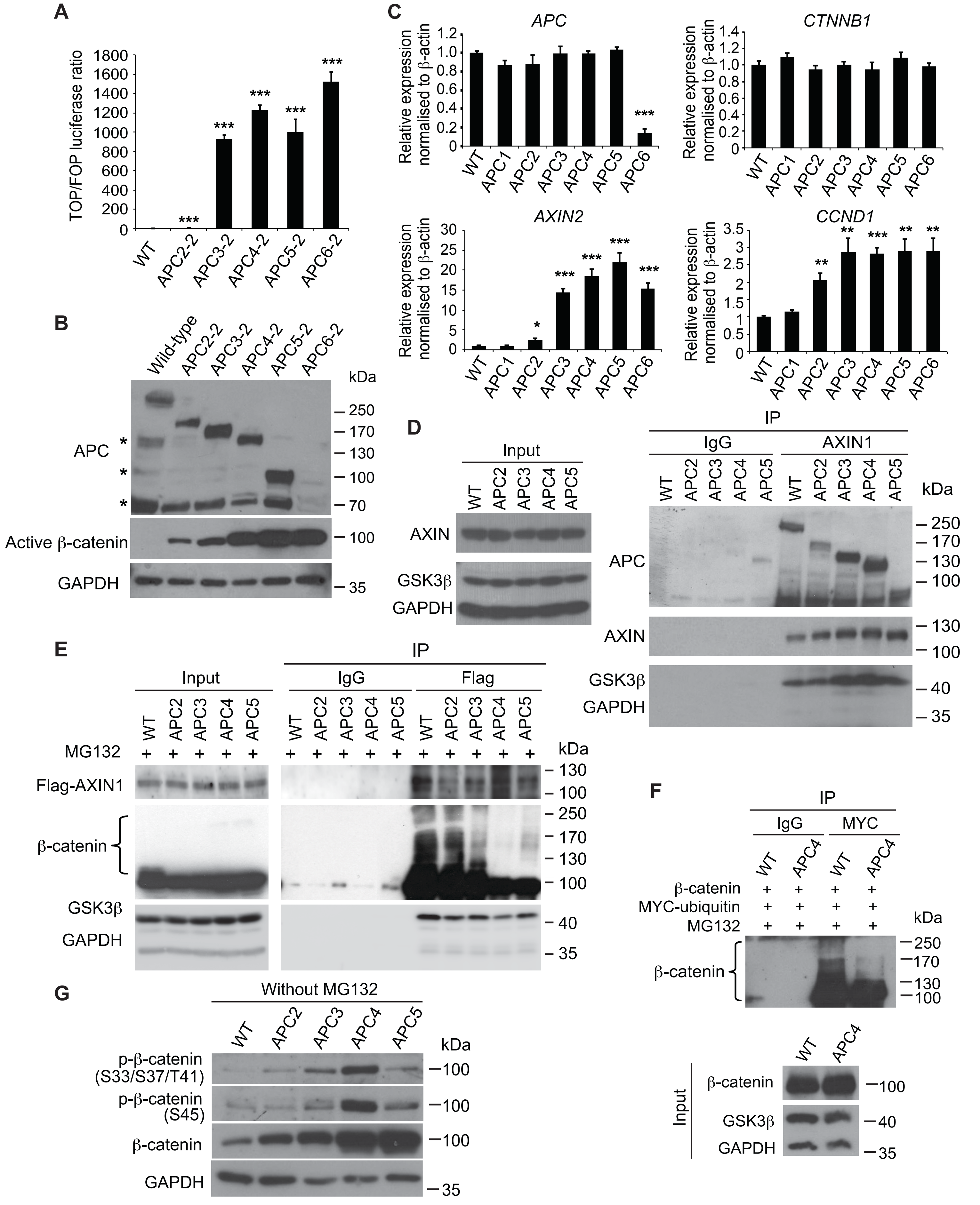

Figure S1, related to Figure 1.

Figure S1, Related to Figure 1. Characterization of the CRISPR-engineered APC truncations. (A) Relative TOP/FOP activities of the HEK293T WT and the second clones of different CRISPR-engineered APC truncated lines. (B) Cell lysates were analyzed by western blotting using the indicated antibodies. (C) mRNA expression of APC, β-catenin (CTNNB1), AXIN2 and CyclinD1 (CCND1) was analyzed by qRT-PCR. Data are presented as fold change normalized to β-actin control in triplicate and are representative of at least three independent experiments. Error bars represent ± standard error (*p<0.05; **p<0.01; ***p<0.001). (D) Cell lysates were immunoprecipitated with AXIN1 antibody and IgG as control followed by western blotting using the indicated antibodies. (E) HEK293T WT and APC truncated cell lines were transfected with Flag-Axin1. Lysates were immunoprecipitated with anti-Flag antibody and IgG as control, followed by western blotting using the indicated antibodies. (F) HEK293T WT and APC4 cell lines were transfected with β-catenin and MYC-ubiquitin. Lysates were immunoprecipitated with anti-MYC antibody and IgG as control, followed by western blotting using the indicated antibodies. (G) Lysates from HEK293T WT and APC truncated cell lines without MG132 treatment were subjected to SDS-PAGE followed by western blot analysis using the indicated antibodies.

A

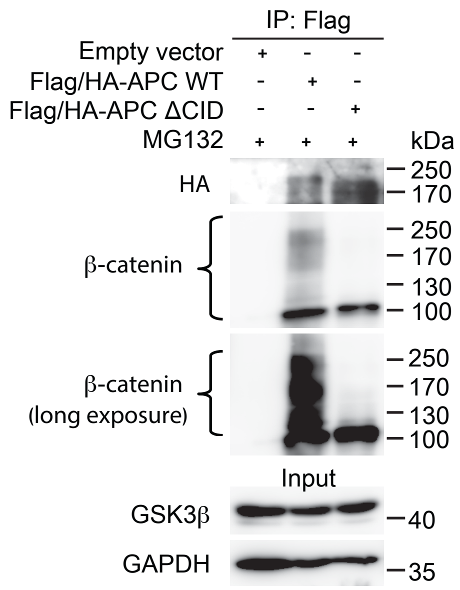

B

Protein names	log10 iBAQ APC WT (293T cells)	log10 iBAQ APC ΔCID (293T cells)	log10 iBAQ APC WT (L cells)	log10 iBAQ APC ΔCID (L cells)	PEP (293T cells)	PEP (L cells)	APC ΔCID Unique peptides (293T cells)	APC ΔCID Unique peptides (Lcells)
APC	7.28	8.38	6.00	7.12	0	0	98	16
CTNNB1	6.00	7.36	5.90	6.96	0	9.93E-284	34	25
CTNNA1	6.55	6.73	5.29	6.36	0	3.53E-175	38	30
CSNK1E	4.54	5.47	5.15	5.15	1.51E-22	3.70E-14	4	4
CSNK1D	3.53	4.69	5.29	5.32	4.69E-11	1.03E-09	2	2
**USP7**	**NaN**	**4.26**	**NaN**	**4.06**	**1.66E-05**	**2.35E-05**	**2**	**1**
USP30	NaN	4.74	--	--	2.11E-08	--	1	--
RNF114	NaN	5.08	--	--	3.66E-17	--	4	--
TRIM21	NaN	4.52	--	--	0.0029507	--	1	--
ZFP91	NaN	4.58	--	--	3.31E-07	--	2	--
RANBP2	NaN	4.53	--	--	1.45E-85	--	9	--
TRIM32	NaN	3.73	--	--	0.0066	--	1	--

C

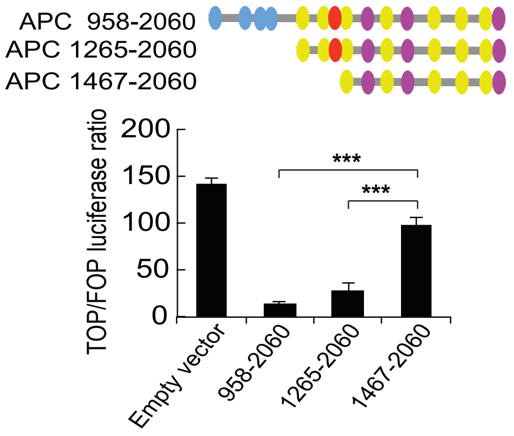

E

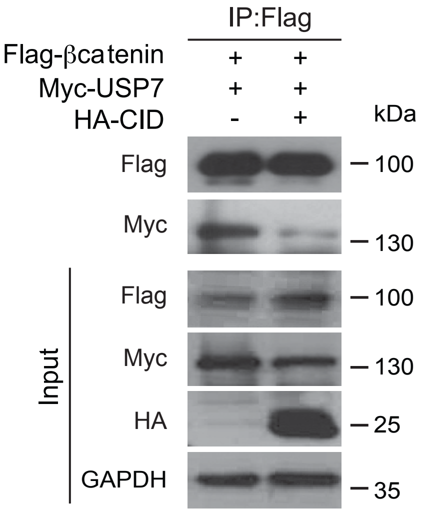

D

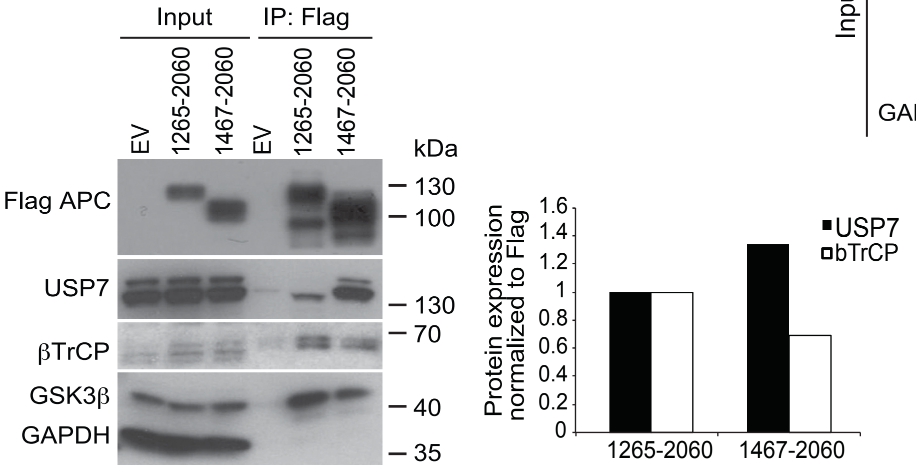

Figure S2, related to Figure 2.

Figure S2, Related to Figure 2. Increased binding of USP7 to the destruction complex upon APC CID loss. (A) HEK293T cells were transfected with empty vector (EV), APC WT, or APC ΔCID. Lysates were immunoprecipitated with anti-Flag antibody followed by western blotting using the indicated antibodies. (B) Protein candidates identified from tandem affinity purification-coupled mass spectrometry in HEK293T and L cells expressing APC WT or APC ΔCID. iBAQ, intensity based absolute quantification; PEP, posterior error probability (p-value for protein identification) (C) Schematic representation of the central region of APC (958-2060) and the indicated deletion constructs. Relative TOP/FOP activities of SW480 cells transfected with the above constructs or empty vector. Error bars represent ± standard error from at least three independent experiments (***$p<0.001$). (D) APC4 cells were transfected with EV and the indicated APC truncated mutants. Lysates were immunoprecipitated with anti-Flag antibody followed by western blot analysis using the indicated antibodies. The graph represents the quantitation of the binding of USP7 and βTrCP in the Flag complexes corresponding to the blot shown in (D). (E) HEK293T cells were transfected with Flag-β-catenin, MYC-USP7 and HA-CID when indicated. Lysates were immunoprecipitated with Flag followed by western blotting using the indicated antibodies.

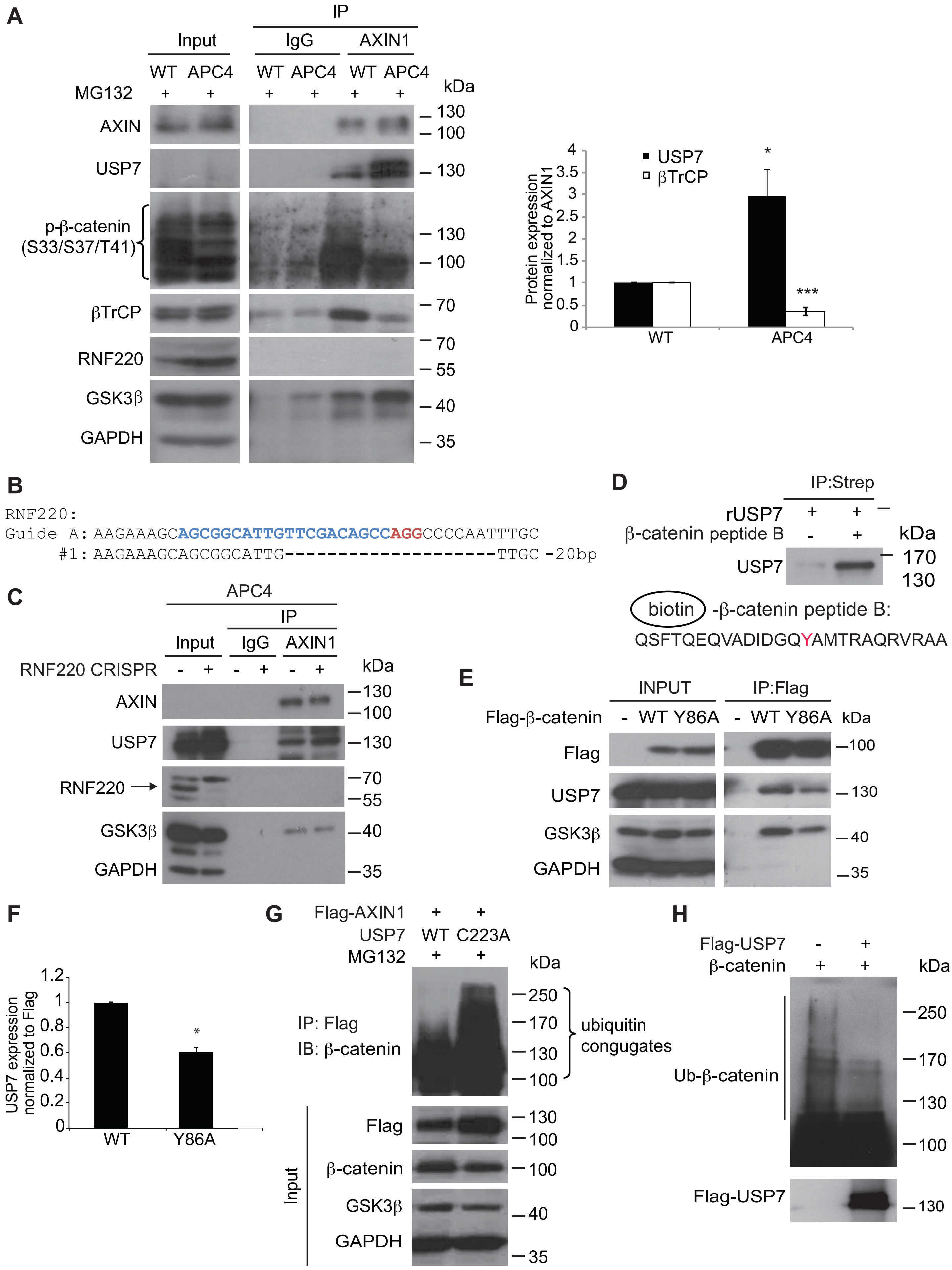

Figure S3, related to Figure 3.

Figure S3, Related to Figure 3. USP7 binding to the β-catenin destruction complex is RNF220-independent. (A) Destruction complexes in HEK293T WT and APC4 cells were immunoprecipitated with AXIN1-specific antibody followed by western blot analysis using the indicated antibodies. The graph represents the amount of USP7 and βTrCP in the Axin1 complexes. Error bars represent ± standard error from at least three independent experiments (*$p<0.05$; ***$p<0.001$). (B) RNF220 sequencing data showing mutation after CRISPR/Cas9 targeting in APC4 cells. (C) APC4 cells with or without RNF220 CRISPR targeting were immunoprecipitated using axin1 antibody. Lysates were analyzed by western blotting using the indicated antibodies. (D) Biotinylated peptide encoding β-catenin peptide B was assayed for binding to recombinant USP7 protein (rUSP7) in streptavidin-based peptide pull-down assays. Modification site Tyr86 is indicated in red. (E) USP7 showed reduced binding to the β-catenin Y86A mutant IP-complex compared to the WT. (F) Quantitation of the USP7 binding in the Flag complexes. Error bars represent ± standard error from at least three independent experiments (*$p<0.05$). (G) HEK293T cells were co-transfected with Flag-Axin1 and USP7 WT or C223A mutant. Lysates were immunoprecipitated with anti-Flag antibody followed by western blot analysis using the indicated antibodies. (H) In vitro deubiquitination assay for β-catenin. Purified ubiquitinated β-catenin was incubated with or without purified Flag-USP7 and analysed by immunoblotting with antibodies for β-catenin and Flag.

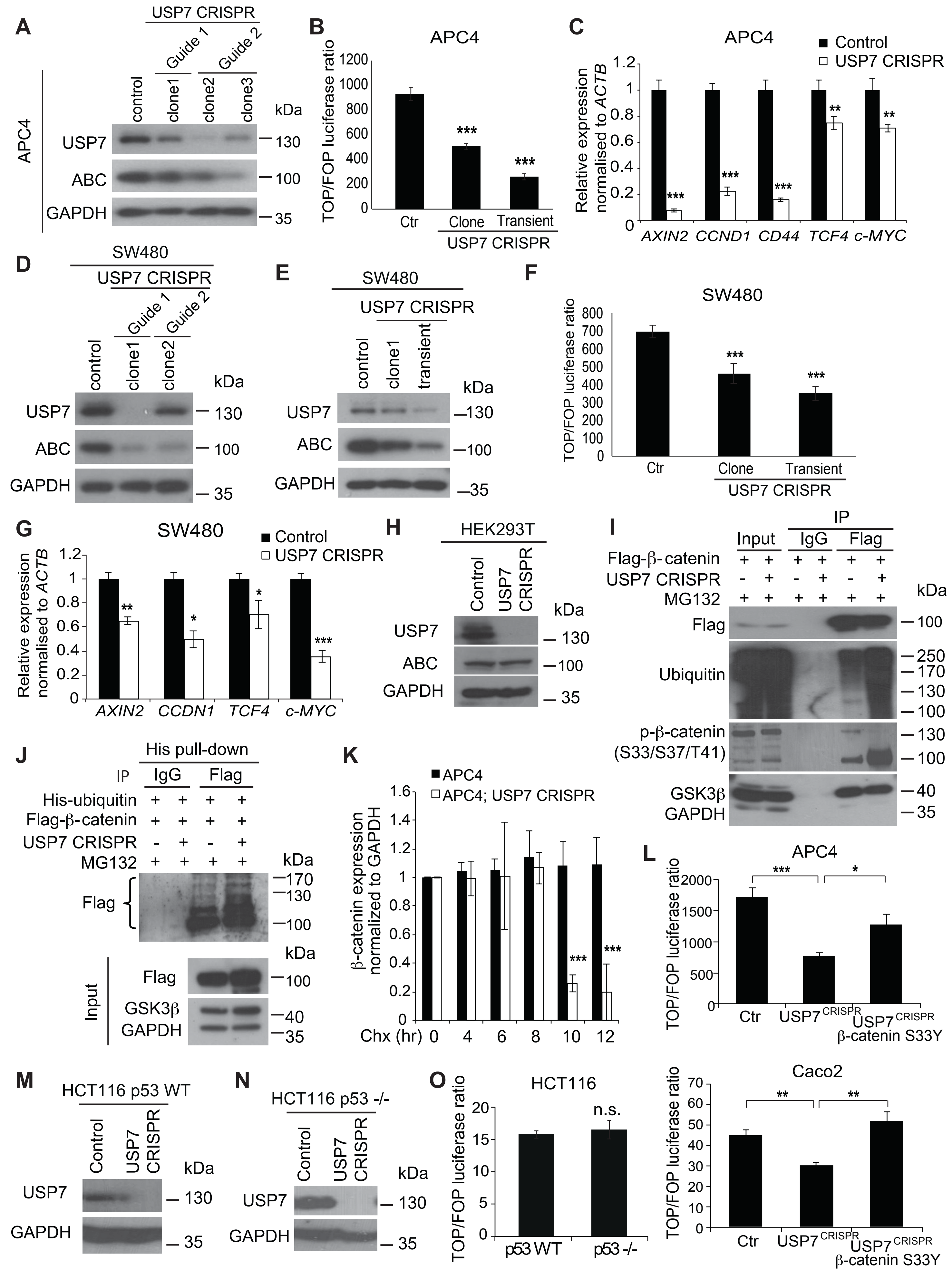

Figure S4, related to Figure 4.

Figure S4, Related to Figure 4. USP7 depletion in APC-mutant cells suppresses aberrant Wnt activation. (A) Western blot analysis of APC4 cells with USP7 CRISPR targeting using antibodies against USP7, active β-catenin (ABC) and GAPDH loading control. (B) Relative TOP/FOP activities of APC4 cells with transient USP7 CRISPR targeting or stable CRISPR clone. Error bars represent ± standard error from at least three independent experiments (***$p<0.001$). (C) mRNA expression of AXIN2, CyclinD1 (CCND1), CD44, TCF4 and c-MYC was analyzed by qRT-PCR in APC4 cells control or USP7 CRISPR. Data are presented as fold change normalized to β-actin (ACTB) control from at least three independent experiments. Error bars represent ± standard error (**$p<0.01$; ***$p<0.001$). (D) Western blot analysis of SW480 with or without USP7 CRISPR targeting using the indicated antibodies. (E) Western blot analysis of the parental SW480, SW480 USP7 CRISPR stable clone or SW480 with transient CRISPR-targeting of USP7 using the indicated antibodies. (F) Relative TOP/FOP activities of the cells in (E). Error bars represent ± standard error from at least three independent experiments (***$p<0.001$). (G) mRNA expression of AXIN2, CyclinD1 (CCND1), TCF4 and c-MYC was analyzed by qRT-PCR in SW480 cells control or USP7 CRISPR. Data are presented as fold change normalized to β-actin (ACTB) control from at least three independent experiments. Error bars represent ± standard error (*$p<0.05$; **$p<0.01$; ***$p<0.001$). (H) Western blot analysis of parental HEK293T and USP7 CRISPR-mutant clone using the indicated antibodies. (I) IP-coupled immunoblotting of transfected flag-βcatenin in APC4 cells with or without USP7 CRISPR-targeting using the indicated antibodies. (J) APC4 cells with or without USP7 CRISPR-targeting were co-transfected with His-Ubiquitin and Flag-β-catenin. Cell lysates were then used for immunoprecipitation with Flag antibody or control IgG as indicated. Flag and IgG complexes were eluted and used in His pull-down assays. Samples were analyzed by western blotting with the indicated antibodies. (K) Quantitation of β-catenin expression normalized to GAPDH in APC4 with or without USP7 CRISPR and treated with cycloheximide (Chx) for the indicated times as shown in Figure 4E. Error bars represent ± standard error from at least two independent experiments (***$p<0.001$). (L) Relative TOP/FOP activities of APC4 (top) and Caco2 cells (bottom) when transfected with the indicated plasmids. Error bars represent ± standard error from at least two independent experiments (*$p<0.05$; **$p<0.01$;***$p<0.001$). (M) Western blot analysis of HCT116 p53 WT cells with or without USP7 CRISPR-targeting using the indicated antibodies. (N) Western blot analysis of HCT116 p53 -/- cells with or without USP7 CRISPR-targeting using the indicated antibodies. (O) Relative TOP/FOP activities of the HCT116 p53 WT compared to HCT116 p53 -/- cells.

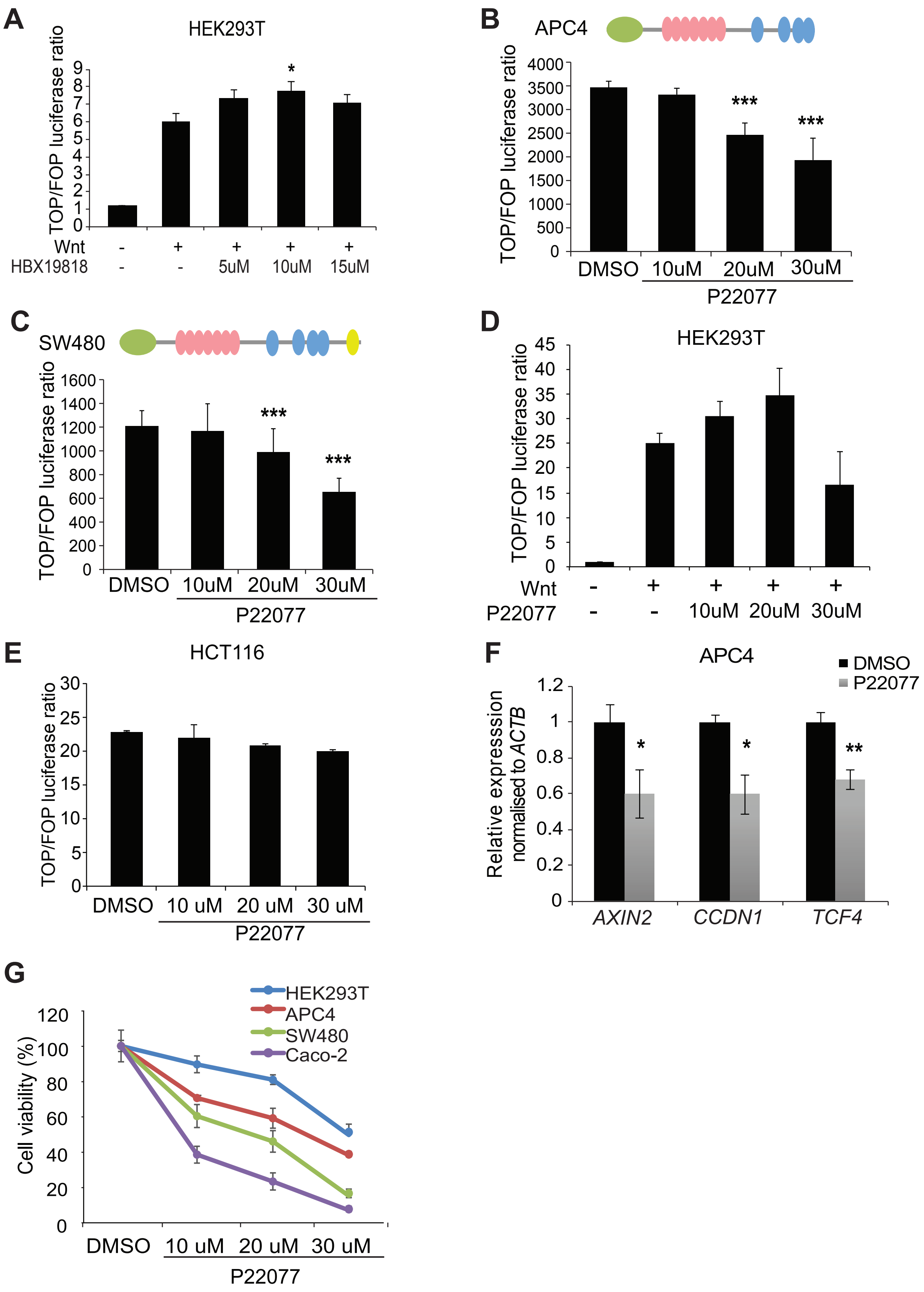

Figure S5, Related to Figure 5. Small molecule inhibition of USP7 in different APC-mutant cell lines. (A) Relative TOP/FOP activity of HEK293T cells treated with DMSO or HBX19818 at the indicated concentrations. Error bars represent ± standard error from at least three independent experiments. (b-e) Relative TOP/FOP activities of APC4 (B), SW480 (C), HEK293T (D) and HCT116 (E) cells treated with DMSO or P22077 at the indicated concentrations. Error bars represent ± standard error from at least three independent experiments. (F) mRNA expression of the indicated Wnt target genes was analyzed by qRT-PCR in APC4 cells treated with DMSO or 10 uM P22077. Data are presented as fold change normalized to β-actin control in triplicate and are representative of at least three independent experiments. (G) MTT assay in the indicated cell lines treated with DMSO or P22077 at the indicated concentrations for 24 hours. Error bars represent ± standard error from at least three independent experiments (*$p<0.05$; **$p<0.01$; ***$p<0.001$)

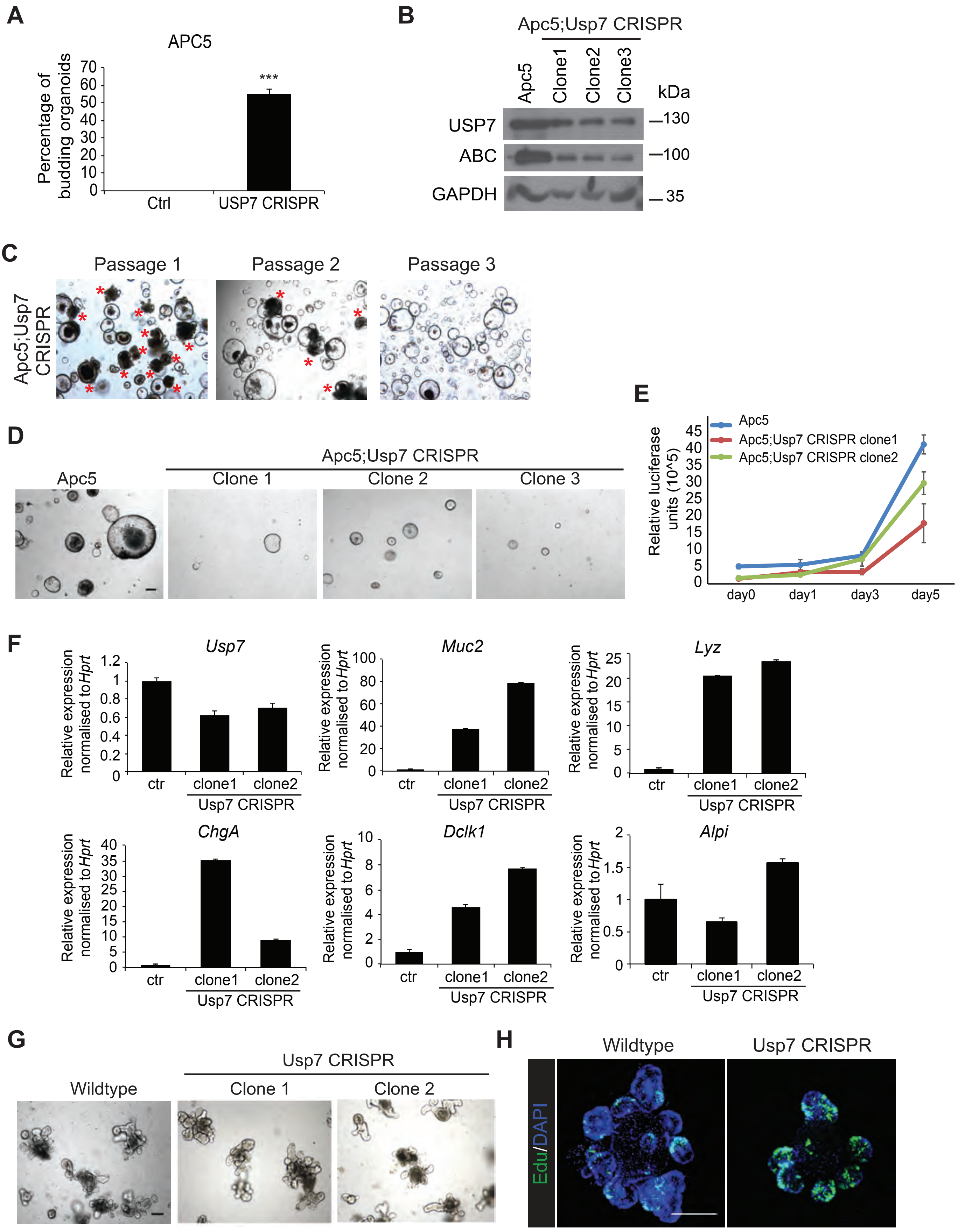

Figure S6, related to Figure 6.

Figure S6, Related to Figure 6. Usp7 deletion in intestinal organoids. (A) Percentage of budding organoids in APC5 after USP7 CRISPR. Error bars represent ± standard error from at least three independent experiments (***p<0.01). (B) Western blot analysis of Apc5 organoids and the individual clones with Usp7 CRISPR-targeting using the indicated antibodies. (C) Representative photos of Apc5 Usp7 CRISPR organoids after several passages. Asterisks indicate budding organoids. Scale bar, 100 µm. (D) Representative photos of colony formation assay in Apc5 organoids and the individual Usp7 CRISPR-clones after 7 days in culture. Scale bar, 100 µm. (E) Cell Titer Glo luciferase assay of the indicated organoids at the indicated times. Error bars represent ± standard error from at least three independent experiments. (F) qRT-PCR analysis of the mRNA expression of Usp7 and the differentiation markers of goblet cells (Mucin2 (Muc2)), Paneth cells (Lysozyme (Lys)), endocrine cells (Chromogranin A (ChgA)), tuft cells (Doublecortin-like kinase 1 protein (Dclk1)) and enterocytes (alkaline phosphatase (Alpi)). Data are presented as fold induction normalized to Hrpt1 housekeeping control in triplicates from one representative experiment. Error bars represent ± standard error. (G) Representative photos of WT organoids and the USP7 deleted clones. Both number and size of spheres formed were significantly smaller in the Usp7-targeted Apc5 organoids. Scale bar, 100 µm. (H) Immunostaining for proliferating cells after 1-hour 5-ethynyl-2′-deoxyuridine (EdU) incorporation.

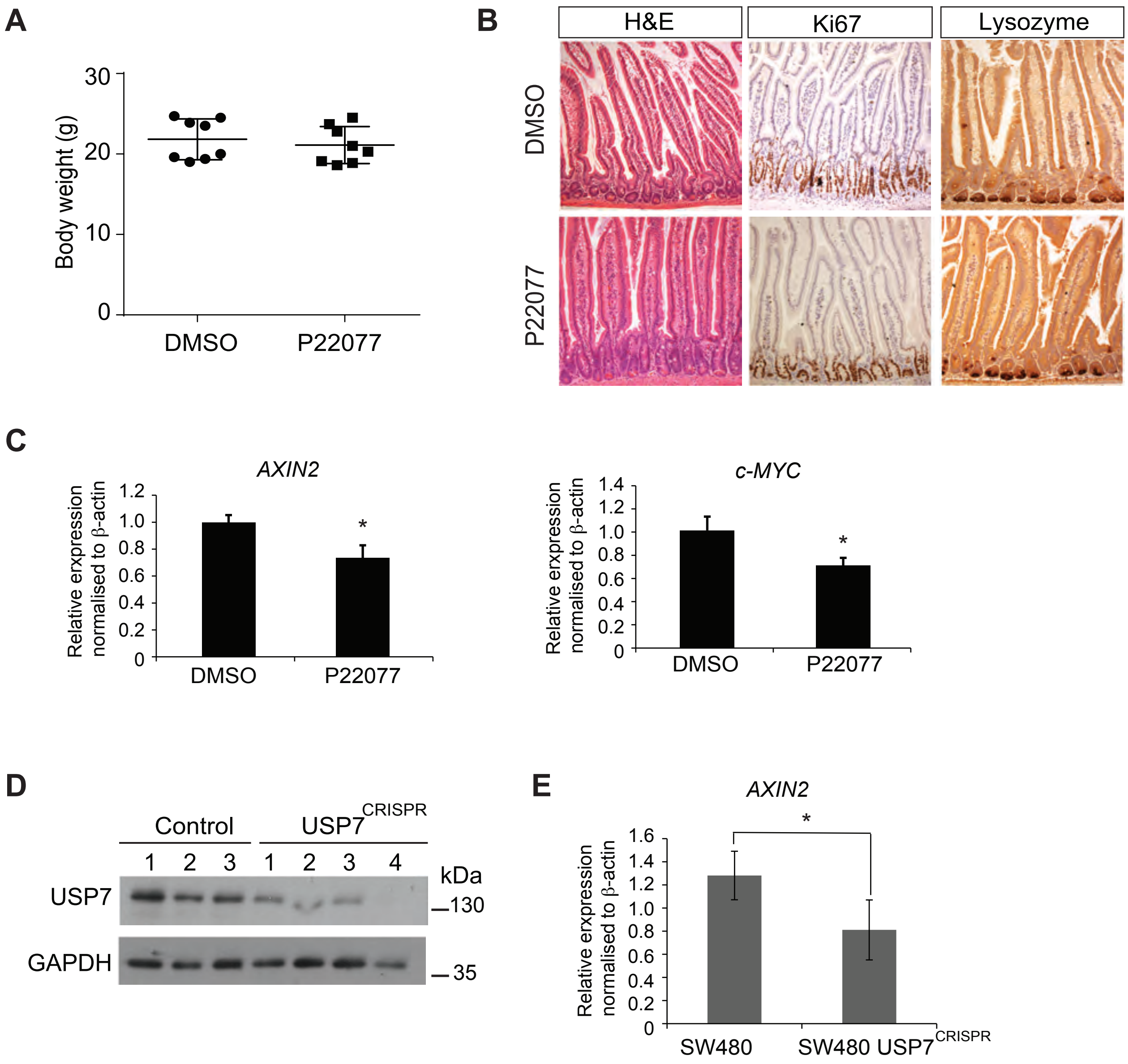

Figure S7, Related to Figure 7. Inactivation of USP7 in SW480-derived xenograft tumors in vivo. (A) Measurement of body weight from mice injected with SW480 treated with DMSO or P22077 inhibitor (30 mg/kg) at the end of the experiment (21 days). (B) Immunohistochemical analysis with Hematoxylin and eosin (H&E), marker of proliferation (Ki67) and Paneth cell marker (Lysozyme) in the intestines harvested from mice treated with DMSO or P22077 inhibitor at the end of the experiment (21 days). (C) mRNA expression of the Wnt target genes AXIN2 and c-MYC in the DMSO- or P22077-treated tumors was analyzed by qRT-PCR. Data is presented as fold induction normalized to β-actin control in triplicate with n=8 per condition. Error bars represent ± standard error (*p,0.05). (D) Western blot analysis of tumors derived from parental SW480 and SW480 USP7CRISPR cells at the end of the experiment using USP7 antibody and GAPDH loading control. (E) mRNA expression of the Wnt target AXIN2 was analyzed by qRT-PCR from the remaining tumor materials described in (D). Data is presented as fold induction normalized to β-actin housekeeping control in triplicate with n=4 per condition. Error bars represent ± standard error (***p<0.001)..

Table S1, Related to Figure 1. Genomic DNA sequencing results depicting mutations in human APC.

APC1

Wt: ACCAAGTCAGCTG**CCTGTGTACAAACTTCTACCATC**ACAAAACAGGTTGCAACCCC

gRNA 1
ACCAAGTCAGCT-----------AACTTCTACCATCACAAAACAGGTTGCAACCCC **-11 bp**
ACCAAGTCAGCTGCCTGTGTAC----------CATCACAAAACAGGTTGCAACCCC **-10 bp**
---------------------------------ATCACAAAACAGGTTGCAACCCC **-78 bp**

APC2

Wt: AAACTATTGATTCTGAAAAGGA**CCTATTAGATGATTCAGATGATG**ATGATATTGAAATACTAGAAGA

gRNA 1
AAACTATTGATTCTGAAAAGGACCTATTAAGATGATTCAGATGATGATGATATTGAAATACTAGAAGA **+1bp**
AAACTATTGATTCTGAAAAGGACCTATTATGATGATTCAGATGATGATGATATTGAAATACTAGAAGA **+1bp**

Wt: ATACAGAAAGATGTGGAATT**AAGAATAATGCCTCCAGTTCAGG**AAAATGACAATG

gRNA 2
ATACA-------------------------------------GAAAATGACAATG **-37 bp**
ATACAGAAAGATGTGGAATTAAGAATAATGCCTCCAG------AAAATGACAATG **-6 bp**

APC3

Wt: ATAGTTTTGAGAGTCGTTCGATTG**CCAGCTCCGTTCAGAGTGAACCA**TGCAGTGGAATGGTAAGT

gRNA 1
ATAGTTTTGAGAGTCGTTCGATTGCCAGCT------AGAGTGAACCATGCAGTGGAATGGTAAGT **-6 bp with STOP**
ATAGTTTTGAGAGTCGTTCGATTGCCAGCTACCGTTCAGAGTGAACCATGCAGTGGAATGGTAAGT **+1 bp**

Wt: TAAGCCCC**AGTGATCTTCCAGATAGCCCTGG**ACAAACCATGCCACCAA

gRNA 2
TAAGCCCCAGTGATCTTCCAG----------ACAAACCATGCCACCAA **-10 bp with stop codon**

APC4

Wt: GCAGTGAGAATACGTCCA**CACCTTCATCTAATGCCAAGAGG**CAGAATCAGCTCCATCCAAGTTCTGCA

gRNA 1
GCAGTGAGAATACGTCCACACCTTCATCTAATGC--------AGAATCAGCTCCATCCAAGTTCTGCA **-8bp**
GCAGTGAGAATACGTCCACACCTTCATCTAATGCCCAAGAGGCAGAATCAGCTCCATCCAAGTTCTGCA **+1bp**

Wt: AATGCCAAGAGGCAGAATCAGCTCCATCCAAGTTCTGCACAGAG**TAGAAGTGGTCAGCCTCAAAAGG**CTGCCACTTGCAAAGTTTCTTCTA

gRNA 2
AATGCCAAGAGGCAGAATCA---CTTGCAAAGTTTCTTCTA **-53bp**
AATGCCAAGAGGCAGAATCAGCTCCATCCAAGTTCTGCACAGAGTAGAAGTGGTCAGCC-------------ACTTGCAAAGTTTCTTCTA **-13bp**

APC5

Wt: GATTATGTTTTTGACA**CCAATCGACATGATGATAATAGG**TCAGACAATTTTAATACTGG

gRNA 1
GATTATGTTTTTGACACCAATC-----GATGATAATAGGTCAGACAATTTTAATACTGG **-5 bp**
GATTATGTTTTTGACACCAATCG-----------ATAGGTCAGACAATTTTAATACTGG **-11 bp**

Wt: ATAGACAATTTAAGTC**CCAAGGCATCTCATCGTAGTAAG**CAGAGACACAAGCAAAGTCT

gRNA 2
ATAGACAATTTAAGTCCCAAGGACATCTCATCGTAGTAAGCAGAGACACAAGCAAAGTCT **+1 bp**
ATAGACAATTTAAGTCCCAAG----------------AGCAGAGACACAAGCAAAGTCT **-16 bp**

APC6

Wt: TTATTTTTCAGTGCCAGCT**CCTGTTGAACATCAGATCTGTCC**TGCTGTGTGTGTTCTAATGAAA

gRNA 1
TTATTTTTCAGTGCCAGCTCC----GAACATCAGATCTGTCCTGCTGTGTGTGTTCTAATGAAA **– 4bp**
TTATTTTTCAGTGCCAGCTCCTGT-GAACATCAGATCTGTCCTGCTGTGTGTGTTCTAATGAAA **-1 bp**

Wt: TTATTTTTCAGTGCCAGCT**CCTGTTGAACATCAGATCTGTCC**TGCTGTGTGTGTTCTAATG

gRNA 2
TTATTTTTCAGTGCCAGCTCCTGT----------------CCTGCTGTGTGTGTTCTAATG **-16 bp**
TTATTTTTCAGT-------------GAACATCAGATCTGTCCTGCTGTGTGTGTTCTAATG **-13 bp**

Table S2, Related to Figure 4. Genomic DNA sequencing results depicting mutations in human *USP7*.

USP7 CRISPR

Cell line	Allele	Sequence	Mutation
APC4	WT:	GATTCCACGTAAGACAGTTTAAATGATGCAACTACAAACCCCCACATCGTTGTAT	
	#1:	GATTCCACGTAAGACA-----------------------------------GTAT	+35 bp
	#2:	GATTCCACGTAAGACAGTTTAAATGATGCAACTACAAACCCCCACATCGTTGTAT	wt
SW480	WT:	GATTCCACGTAAGACAGTTTAAATGATGCAACTACAAACCCCCACATCGTTGTAT	
	#1:	GATTCCACGTAAGACA-----------------------------------GTAT	+35 bp
	#2:	GATTCCACGTAAGACAGTTTAAATGATGCAACTACAAACCCCCACATCGTTGTAT	wt
HEK293T	WT:	CTTTTATCCAGACAGACCACACCAAAAAA-------------GCGTAGGAT	
	#1:	CTTTTATCCAGACAGACCACACCATCAGATTCTTTCTCCAGTGCATAGGAT	+13 bp
	#2:	CTTTTATCCAGACAGACCACACCAAAAA-----AGGAT	-5 bp
HCT116 p53 WT	WT:	CTTTTATCCAGACAGACCACACCAAAAAAGCGTAGGAT	
	#1:	CTTTTATCCAGACAGACCACACCAAAAA----- AGGAT	-5 bp
HCT116 p53 -/-	WT:	GGATAAAAGCGTGGCATCACCATAATCTTCCATGGCAGATTTCGCACAAAACACG	
	#1:	GGATAAAAGCGTGGCA-CACCATAATCTTCCATGGCAGATTTCGCACAAAACACG	-1 bp

Table S3, Related to Figure 6. Genomic DNA sequencing results depicting mutations in mouse *Apc* and *Usp7*.

Mouse *Apc*

APC2.5

Wt: CAGACT**GCTGTAAATGCTGCCGTGCAGAGG**GTGCAGGTCCTTCCAGACGTGGATACTTTG

CAGACTGCTGTAAATGCTGCCGTG--GAGGGTGCAGGTCCTTCCAGACGTGGATACTTTG **-2 bp**

CAGACTGCTGTAAATGCTGCCGTGAGTCCTGTCGGGTTTCGCCACCTCTGACTTGAGCGTCGATTTTTGTGATACTCGCAGAGGGTGCAGGTCCTTCCAG **+54 bp**

APC3

Wt: **GAGTGAGCCATGTAGTGGAATGG**TGAGTGGCATCATAAGC

GAGTGAGCCATGTAGTGGAATGG------+238------TGAGTGGCATCATAAGC **+250 bp**

APC5

Wt: AATCTCACAGCTTGACAATAGTCA**GTAATGCATGTGGAACTTTGTGG**AATCTCTCAGCA

AATCTCACAGCTTGACAATAGTCAGTAATGCATGTGGAA-TTTGTGGAATCTCTCAGCA **-1 bp**

AATCTCACAGCTTGACAATAGTCAGTAATGCATGTGGAA----------TCTCTCAGCAAGAAATCCTAAAGACCAG **-10 bp**

Mouse *Usp7*

Wild type organoids-USP7 CRISPR

Wt:

AGACA**CCAGTTGGCGCTCCGAGGCAACC**TTTCAGTTCACTGTCGAGCGCTTCAGCAGACTGAGTGAGTCGGTCCTTAGCCCTCCGTGTTTTGTGCGAAATCTGCCATGGAAGATTA
TGGTGATGCCACGCTTTTATCCAGACAGACCACACCAAAAAAGCGTAGGATTCTTTCTCCAGTG

CRISPR targeted:

AGACACCAGTTGGCGCTCCGAGGCAACCTTTCAGTTCACTGTGGAGCGCTTCAGCAGACTGAGTGAGTCGGTCCTTAGCCCTCCGTGTTTTGTGCGAAATCTGCCATGGAAGATTA
TGGTGATGCCACGCTTTTATCCAGACAGACCACACCAAAAA-----AGGATTCTTTCTCCGGTG **-5 bp**

AGACA**CCAGTTGGCGCTCCGAGGCAACC**TTTCAGTTCACTGTCGAGCGCTTCAGCAGACTGAGTGAGTCGGTCCTTAGCCCTCCGTGTTTTGTGCGAAATCTGCCATGGAAGATTA
TGGTGATGCCACGCTTTTATCCAGACAGACCACACCAAAAAAGCGTAGGATTCTTTCTCCAGTG **wt**

APC5 organoids-USP7 CRISPR

Wt: GCTCGACAGTGAACTGAAA**GGTTGCCTCGGAGCGCCAACTGG**TGTCTGCAAGAAAAC

GCTCGACAGTGAACTGAAA**GGTTGCCTCGGAGCGCCAACTGG**TGTCTGCAAGAAAAC **wt**

GCTCGACAGTGAACTGAAAGGTTG-----GAGCGCCAACTGGTGTCTGCAAGAAAAC **-5 bp**

SUPPLEMENTAL EXPERIMENTAL PROCEDURES

Antibodies

APC (Calbiochem), AXIN1 (from the Clevers' lab) (Li et al., 2012), Axin1 (C95H11, Cell Signaling), GAPDH (Santa Cruz), GSK3β (Cell Signaling), phospho-β-catenin S45 (Cell Signaling), phospho-β-catenin S33/S37/T41 (Cell Signaling), β-catenin (BD Transduction), active-β-catenin (Millipore, 8E7), Flag (F3165, SIGMA), HA (Y-11) (Santa Cruz), c-Myc (9E10, Santa Cruz), RNF220 (Sigma), β-TrCP (ITK Diagnostics BV and Cell Signaling), ubiquitin (Millipore, FK2) and USP7 (Bethyl Laboratories) were used in immunofluorescence, immunoprecipitations or Western blot analysis.

Plasmids and other reagents

Full-length USP7 was cloned into XhoI site of pcDNA-Flag or pcDNA-MYC vectors. Various mutants were generated by PCR into pcDNA-Flag or pcDNA-MYC. Mutants include (ΔMath-USP7, ΔCatalytic-USP7, ΔHUBL-USP7 and USP7-C223A). APC overexpression constructs were described in (Li et al., 2012). HBX41108 (Tocris Bioscience) was resuspended in DMSO at 10 mM. DUB inhibitor VI, P22077 (Calbiochem) was resuspended in DMSO at 10 mM (for cell lines experiments) or 15 mg/ml (for mice experiments). Cycloheximide (C7698, SIGMA) was resuspended in ethanol at 50 mg/ml.

His Pull-Down Assay

APC4 cells were transfected with a His-Ubiquitin expression construct for at least 12 hr before treatment with HBX41108 or DMSO for 16 hr (Fig S5E). Cells were lysed in buffer A and continue with His-pull down protocol. Or APC4 and APC4 USP7 CRISPR cells were transfected with His-Ubiquitin and Flag-β-catenin for at least 12 hr (Fig S4J). Lysates were immunoprecipitated using Flag antibody. The immunoprecipitated complexes were washed three times with cold PBS and eluted in buffer A (6M Guanidinium-HCl+ 0.1M Na_2HPO_4+ 0.1M NaH_2PO_4 + 0.01M pH8.0 Tris/HCl + 20mM Imidazole + 10mM β-mercaptoethanol). The eluted supernatants were incubated with buffer A pre-washed Ni^{+2}-NTA agarose beads for 4 hr at room temperature (R.T.). After incubation and centrifugation (4000rpm at 2min at R.T.), the pellets were washed once with buffer A, once with buffer B (8M urea, 0.1 M Na2HPO4/NaH2PO4, 10 mM Tris-HCl (pH 6.8), 0.005 M imidazole, 0.01 M β-mercaptoethanol), twice with buffer B plus 0.1% Triton-X-100. Elute with elution buffer (0.2 M imidazole, 0.15 M Tris-HCl (pH 6.8), 30% glycerol, 0.72 M β-mercaptoethanol, 5% SDS) for 20 min at RT with gentle agitation. The elution was resuspended in 4XSDS loading sample buffer, and boiled for 5 min. The pull-down samples were subjected to SDS-PAGE for Western blot analysis.

In vitro deubiquitination assay

The *in vitro* deubiquitination assay was performed as previously described (Dar et al., 2013). Briefly, HEK293T cells were transfected with Flag-β-catenin, MYC-βTrCP and MYC-Ubiquitin for at least 12 hr before treatment with MG132 (10 μM) for 4hr. Cells were lysed in cold lysis buffer containing 150 mM NaCl, 30 mM Tris (pH 7.5), 1 mM EDTA, 1% Triton X-100, 10% Glycerol, 0.1 mM PMSF, 0.5 mM DTT, protease inhibitor cocktail tablets (EDTA-free) (Roche), and phosphatase inhibitor cocktail tablets (Roche) supplemented with 10 μM MG132. Ubiquitinated β-catenin was purified from the cell extracts using anti-Flag M2 affinity beads (Sigma) and washed extensively. Flag-USP7 was transfected into HEK293T cells and purified using anti-Flag M2 affinity beads (Sigma), which was then eluted with Flag-peptides (SIGMA). For the *in vitro* deubiquitination assay, purified ubiquitinated β-catenin was incubated with or without purified Flag-USP7 in a deubiquitination buffer (50 mM Tris-HCl pH 8.0, 50 mM NaCl, 1 mM EDTA, 10 mM DTT, 5% glycerol) at 37°C for 1hr 30 min. Reactions were terminated by the addition of 4X SDS loading sample buffer and analysed by immunoblot.

Tandem affinity purification (TAP)-coupled mass spectrometry

Flag- and HA-tagged APC wild type (WT) or APC mutant (ΔCID) constructs were transfected to human HEK293T or mouse L-cells followed by TAP as previously described (Nakatani and Ogryzko, 2003). Eluted proteins were separated by SDS-PAGE and eight bands covering the entire lane were excised for each sample. In-gel trypsin digestion was performed using a Perkin Elmer Janus liquid handling system. The peptide samples were subjected to LC-MS/MS analysis using a LTQ-Orbitrap Velos instrument for data acquisition. Raw spectra were

processed using the MaxQuant/Andromeda bioinformatics suite 1.3.0.5 (Cox and Mann, 2008) and further analyzed in Perseus 1.4.0.0. Data was searched against Uniprot fasta databases containing human/mouse sequences. Intensity based absolute quantification (iBAQ) (Schwanhausser et al., 2011) was used for label free quantification. APC WT and APC ΔCID were compared in human HEK293T and mouse L-cells to screen for changing interactors. The results were filtered as follows. The original dataset contained 778 human proteins and 997 mouse proteins. Only proteins that received exclusive iBAQ values in the APC ΔCID and APC WT were considered as candidates. This reduced the dataset to 268 human proteins and 88 mouse proteins. Further screening of E3 ubiquitin-ligases and DUB enzymes resulted in 7 protein candidates (USP7, UPS30, RNF114, TRIM21, ZFP91, RANBP2 and TRIM32) that showed differential binding between APC WT versus ΔCID complexes.

Peptide Array

Peptide arrays for β-catenin were made from 26 mer peptides derived from β-catenin. Starting from the N terminus, each 26 mer peptide in the array was advanced from the previously by 1 residue in the C-terminal direction. Peptides were arrayed on cellulose membranes (the Crick Peptide Chemistry Platform) and activated using methanol following the protocol described in (Frank and Dubel, 2006). Membranes were washed with TBS three times for 10 min and incubated overnight in 10 ml MBS. Then the membranes were washed once in TBS 0.05%Tween (T-TBS) for 10 minutes and incubate for 2-4 hours in the presence or absence of USP7 recombinant protein (11681-H20BL-25, Sino Biological) sealed in a plastic bag. The membranes were then washed three times in 10 ml of T-TBS (for 10 minutes each) and incubated 1-2 hours with secondary antibody diluted in MBS. After further washes, spots with bound USP7 were detected using anti-rabbit IgG-HRP-conjugated secondary antibody and visualized by chemiluminescence.

Peptide Synthesis and Pull-Down Experiments

The peptide pull-down was carried out using the following biotinylated peptides generated by the Crick Peptide Chemistry Platform: β-catenin peptide A, Bio-SYLDSGIHSGATTTAPSLSGKGNPEE; β-catenin peptide B, Bio-QSFTQEQVADIDGQYAMTRAQRVRAA. 2 μg of peptide A and 7 μg of peptide B were coupled to 40 μl of Streptavidin-coated magnetic beads (Invitrogen). The coupled beads were then incubated with 100 ng of recombinant USP7 (11681-H20BL-25, Sino Biological) in binding buffer (50 mM Tris-HCl pH 7.5, 300 mM NaCl, 0.1% Nonidet NP-40) at 4°C for 2hours. The beads were washed with TTBS (Tris buffer saline, 0.1% Tween20) and resuspended in 4XSDS loading sample buffer, and boiled for 5 min.

Gene Editing with CRISPR/Cas9 System

To generate APC truncations, HEK293T cells were transfected with plasmids encoding Cas9 (#41815, Addgene) modified to add puromycin resistance and guideRNAs (gRNAs) (gRNA-GFT-T1 was, #41819, Addgene). Both Addgene plasmids were a kindly gift from George Church (Mali et al., 2013). The GFP targeting sequence was exchanged by inverse PCR followed by DpnI digestion and T4 ligation. Specifically, a common forward primer 5'-phospho-GTTTTAGAGCTAGAAATAGCAAGTTAAAATAAGG-3' was used in combination with target specific reverse primers (i.e. reverse complement target sequences without the PAM were fused to the 5' end of a common reverse oligo 5'-CGGTGTTTCGTCCTTTCCACAAGAT-3') to generate pU6-sgRNA by using the high-fidelity Phusion polymerase (NEB) on the gRNA-GFT-T1. These gRNAs were targeting specific genomic loci. Guide RNA sequences for APC targeting were shown in Table S1. For APC4 and SW480, two USP7 guide RNAs were used: gRNA1, 5'-ATCATTTAAACTGTCTTACG-3' and gRNA2: 5'-TTAGTCTAAGTCGTAACGTG-3'. For SUP7 targeting in HEK293T and HCT116 cells, either gRNA1, 5'-AGACCACACCAAAAAAGCGT-3' or gRNA2 5'GGCATCACCATAATCTTCCA-3' were used. SW480 or HCT116 cells were transfected with plasmids encoding Cas9 and gRNAs (PX459, #62988, Addgene which was a gift from Feng Zhang lab) (Ran et al., 2013). We screened the potential targeted cells by immunoblotting and then confirmed by genomic DNA sequencing.

Real-time quantitative RT-PCR

RNA was extracted according to the manufacturer's instructions (Qiagen RNAeasy). cDNA was prepared using Maxima first strand cDNA synthesis kit with dsDNase (#1672, Thermo Scientific). Quantitavie PCR detection was performed using iTaq SYBR Green Supermix (#172-5121, Bio Rad) using specific primers to: hUSP7: F: 5' TATGGGGAGTGAAGTGACCGAT 3' R: 5' CATGTACACAGCCTTTCGTAGC 3'; hβ-actin F: 5' TTCTACAATGAGCTGCGTGTG 3' R: 5' GGGGTGTTGAAGGTCTCAAA 3'; hCyclin D1: F: 5' CTCCGCCTCTGGCATTTTGG 3' R: 5' TCTCCTTGCAGCTGCTTAG 3'; hCD44: F: 5'

AGAAGGTGTGGGCAGAAGAA 3' R: 5' AAATGCACCATTTCCTGAGA 3'; hAxin2: F: 5' AGTGTGAGGTCCACGGAAAC 3' R: 5'CTTCACACTGCGATGCATTT 3'; hAPC: F: 5' GAGAGTGCAGTCCTGTTCCT 3' R: 5' GTGAGATTCTGAAGTTGAGCGT 3'; hCTNNB1: F: 5' ACCTTTCCCATCATCGTGAG 3' R: 5' AATCCACTGGTGAACCAAGC 3'; *mUSP7*: F: 5' TGCTGAATCTGACTCCACGT 3' R: 5' CCCAGTCGTTTTCCTTGTGG 3'; *mLys* F: AAAGGAATGGAATGGCTGGC 3' R: 5' TCGGTTTTGACAGTGTGCTC 3'; *mChgA* F: 5' GCCACCAATACCCAATCACC 3' R: CCTCCTCTTCCTCCTCCTCT 3'; *mMucin2* F: 5' TCTACCTCACCCACAAGCTG 3' R: 5'TGGTCTGCATGCCATTGAAG 3'; *mAlpi* F: 5' TCAACGAGATCCCCTGATGG 3' R: 5'TATGGTCAGAGTGTCGCGTT 3'; *mDclk1* F: 5' CGCTTCAGATCTTTCGAGGC 3' R: 5' CCGCAGACATAGCTTTCACC 3'; *mHrpt1*: F: 5'TCATGAAGGAGATGGGAGGC 3' After cDNA amplification (40 cycles), samples were normalized to β-actin (human) or *Hrpt1* (mouse), and data were expressed as mean ± SD.

Immunofluorescence

HEK293T cells were grown on 12-well coverslips in 12-well plates precoated with poly-L-lysin (Sigma), fixed with 4% paraformaldehyde (PFA) for 15 min, and permeabilized by 0.2% Triton X-100 in PBS for 10 min. Cells were blocked with 0.5% BSA in PBS for 1h and followed by primary anti-β-catenin antibody (BD Biosciences) incubation at 4°C overnight. Cells were washed three times with PBS and incubated with the secondary antibodies conjugated to Alexa-Fluor 488 at room temperature for 1h. Cells were washed three times with PBS and stained with DAPI for 10 min. Coverslips were washed another three times with PBS and were then mounted with Aqua Poly/Mount (Polysciences).

Immunohistochemistry

For analysis of small intestine and colon by immunohistochemistry, tissues were fixed in 10% formalin and embedded in paraffin. Sections were deparaffinized with xylene and rehydrated in a graded series of ethanol. Antigen-retrieval was performed for 20 min at high temperature in citrate buffer. Then, slides were blocked and incubated overnight with anti-ki67 (SP6, Abcam) anti-β-catenin (BD, Biosciences) or negative control at 4C. Finally, slides were incubated with the secondary antibody for 1h, washed three times with PBS and incubated with peroxidase substrate, and mounted. The expression of Ki67 or b-catenin was visualized using a bright-field microscope.

MTT assay

MTT assay was used to monitor cell viability after P22077 and HBX19818 treatment in cells using MTS Reagent Powder (G111B, Promega) as described in (Mosmann, 1983).

Colony formation assay

SW480 and HEK293T cells were trypsinized and counted prior seeding. 2000 cells per condition were seeded in 6-well plates and cultured for 15 days. The plates were then fix with 4%PFA for 5 minutes followed by crystal violet staining. Number of colonies formed in each well was then counted. Experiments were performed in triplicates

Intestinal organoid culture

Organoids were established from freshly isolated wild type small intestine or tumors isolated from Apc^{min} mice. Tissues were incubated in cold PBS containing 2mM EDTA for isolating epithelial crypts and cultured as previously as described (Sato et al., 2009) except that Matrigel was replace with Cultrex® BME, Type 2 RGF PathClear (Amsbio, 3533-010-02). In brief, the organoid basal media contains EGF (Invitrogen PMG8043), Noggin and R-spondin (ENR). Noggin and R-spondin conditioned media (CM) were generated from HEK293T cells. Wnt3a CM was generated from L cells. The Rho kinase inhibitor Y-27632 (Sigma) was added to the culture when trypsinsed.

Organoid imaging

Organoids were grown in 20 µl of RGF BME into an 8-well chamber side (Lab-Tek II, 154534). When indicated, 10 µM EdU was added to the growth media for 2 hours before fixing. Organoids were fixed in formalin for 30 minutes. They were then permeabilized in 0.8% Triton for 20 minutes and blocked in IF Buffer (0.2% Triton, 0.05% Tween, 1% BSA, PBS) for 1 hour or processed for EdU staining according to manufacturer's instruction with the Click-iT Edu Alexa Fluor 647 Imaging kit (Invitrogen C10340). For immunofluorescent staining, cells were incubated with primary antibodies overnight in IF buffer: rabbit anti-KRT20 (1:200, Cell Singling Technologies, #13063), rabbit anti-Mucin2 (1:600, Santa Cruz, H-300, sc-1534), anti-Lysozyme (1:600, Dako, #EC3.2.1.17). They were then washed 3 times with TBS-1% Tween and secondary antibodies (1:1000 in IF

Buffer) were incubated for 1h at room temperature. When indicated, organoids were washed 3 times with TBS-1% Tween and were incubated with Phalloidin-FITC (SIGMA, P5282) for 1 hour at 4°C. Then the solution was removed and DAPI was incubated in IF buffer for 10 minutes and washed three times with TBS-1% Tween. The chambers were then removed and cover slips were mounted using Prolong Gold antifade medium (Invitrogen P36930). Images were acquired using Leica Confocal DM 2500 and the Leica Application Suite software. Images were processed using FIJI (Image J) and Photoshop CS5 software (Adobe Systems Inc, San Jose, Ca).

CRISPR/Cas9-gRNA lentivirus production
HEK293T were seeded in a 15 cm plate at high confluence. Before transfection, the media was changed to DMEM without FBS. Cells were then transfected with 60 µg of lentiCRISPR (#52961, Addgene which was a kindly gift from Feng Zhang lab) (Sanjana et al., 2014), 50 µg Pax2 and 16.6 µg VSVG using OPTI-MEM (GIBCO) and PEI (1mg/ml) 1:3 (PEI: DNA) ratio. After 6 hours, the supernatant was discarded and changed to DMEM plus 10% FBS. 48 hours later, the media was collected and filtered through 0.45 µm filter and centrifuged at 20 000 rpm for 1:40 hours at 4°C. The pellet was then resuspended in 250 µl of DMEM Basal Media (Advanced DMEM F/12 containing Pen/Strep, Glutamine and HEPES) and added directly to the trypsinized organoids as concentrated virus or froze at -80°C.

CRISPR targeting of intestinal organoids
Mouse organoids were CRISPR targeted using lentiCRISPR v2 which was a kindly gift from Feng Zhang (#52961, Addgene). The cloning protocol used is described: http://genome-engineering.org/gecko. Two different guideRNAs were used to target USP7 in organoids. gRNA1: 5'-TGCCTCGGAGCGCCAACTGG-3' and gRNA2: 5'-CTGAATCTGACTCCACGTCG-3'. Mouse organoids were cultured in ENR media plus Nicotinamide and Wnt3a CM prior CRISPR-targeting to enrich for stem cells. Before infection, organoids were trypsinized and resuspended in ENR medium plus Nicotinamide, Wnt3a CM, Rho kinase inhibitor Y-27632 and polybrin (8 µg/ml). The resuspened organoids were then mixed with the concentrated virus supernatant and seeded in 48 well plates at high density. The plate was centrifuged at 600 g at 32 °C for 1 hour, and incubated for 4 hours at 37°C before replating in BME with growth medium plus Y-27632. Growth medium was then switched to selection medium containing 2 µg/ml puromycin 3 days post infection.

Cell Titer Glo
Organoids were trypsinized and counted. 2000 single cells were seeded in BME per 48 well and placed in a 37°C incubator to polymerize for 20 minutes. 300 µl of small intestinal organoid growth media (see above) plus Y-27632 was then added and cultured for the indicated times. Cell titer Glo Luminiscent Cell viability assay (G7572, Promega) was used to assess viability of the organoids.

Organoid colony formation assay
Organoids were trypsinized and counted. 2000 single cells were seeded in BME per 48 well and placed in a 37°C incubator to polymerize for 20 minutes. 300 µl of small intestinal organoid growth media (see above) plus Y-27632 was then added and cultured for 7 days. Number of spheres formed in each well was counted as plating efficiency. Experiments were performed in quadruplicate.

Xenograft experiments
5×10^6 human SW480 or HCT116 cells were subcutaneously injected into both flanks of 6-8-week-old SCID mice. For the USP7 inhibitor experiments, SW480-derived xenografts were allowed to grow 2-3 mm before randomizing the mice into a control group (DMSO) or P22077 (30 mg/kg) by intraperitoneal injection every day for 21 days. At the end of the experiments, all mice were killed. Tumors were collected, weighted, and photographed and were freeze with liquid nitrogen till they were processed for RNA or protein extraction. All mice were housed in a pathogen-free environment and handled in strict accordance to institutional protocol.

Supplemental References

Cox, J., and Mann, M. (2008). MaxQuant enables high peptide identification rates, individualized p.p.b.-range mass accuracies and proteome-wide protein quantification. Nat Biotechnol *26*, 1367-1372.
Dar, A., Shibata, E., and Dutta, A. (2013). Deubiquitination of Tip60 by USP7 determines the activity of the p53-dependent apoptotic pathway. Mol Cell Biol *33*, 3309-3320.
Frank, R., and Dubel, S. (2006). Analysis of protein interactions with immobilized Peptide arrays synthesized on membrane supports. CSH Protoc *2006*.

Li, V. S., Ng, S. S., Boersema, P. J., Low, T. Y., Karthaus, W. R., Gerlach, J. P., Mohammed, S., Heck, A. J., Maurice, M. M., Mahmoudi, T., and Clevers, H. (2012). Wnt signaling through inhibition of beta-catenin degradation in an intact Axin1 complex. Cell *149*, 1245-1256.

Mali, P., Yang, L., Esvelt, K. M., Aach, J., Guell, M., DiCarlo, J. E., Norville, J. E., and Church, G. M. (2013). RNA-guided human genome engineering via Cas9. Science *339*, 823-826.

Mosmann, T. (1983). Rapid colorimetric assay for cellular growth and survival: application to proliferation and cytotoxicity assays. J Immunol Methods *65*, 55-63.

Nakatani, Y., and Ogryzko, V. (2003). Immunoaffinity purification of mammalian protein complexes. Methods Enzymol *370*, 430-444.

Ran, F. A., Hsu, P. D., Wright, J., Agarwala, V., Scott, D. A., and Zhang, F. (2013). Genome engineering using the CRISPR-Cas9 system. Nat Protoc *8*, 2281-2308.

Sanjana, N. E., Shalem, O., and Zhang, F. (2014). Improved vectors and genome-wide libraries for CRISPR screening. Nat Methods *11*, 783-784.

Sato, T., Vries, R. G., Snippert, H. J., van de Wetering, M., Barker, N., Stange, D. E., van Es, J. H., Abo, A., Kujala, P., Peters, P. J., and Clevers, H. (2009). Single Lgr5 stem cells build crypt-villus structures in vitro without a mesenchymal niche. Nature *459*, 262-265.

Schwanhausser, B., Busse, D., Li, N., Dittmar, G., Schuchhardt, J., Wolf, J., Chen, W., and Selbach, M. (2011). Global quantification of mammalian gene expression control. Nature *473*, 337-342.
